# Magnetically modified-mitoxantrone mesoporous organosilica drugs: an emergent multimodal nanochemotherapy for breast cancer

**DOI:** 10.1186/s12951-024-02522-4

**Published:** 2024-05-14

**Authors:** Eva Romaní -Cubells, Samuel Martínez-Erro, Victoria Morales, Ana Chocarro-Calvo, José M. García-Martínez, Raúl Sanz, Custodia García-Jiménez, Rafael A. García-Muñoz

**Affiliations:** 1 Department of Chemical and Environmental Technology, Rey Juan Carlos University (URJC), C/Tulipán s/n, Móstoles, Madrid, 28933 Spain; 2 Department of Basic Health Sciences, Rey Juan Carlos University (URJC), Avda. Atenas s/n, Alcorcón, Madrid, 28922 Spain

**Keywords:** Periodic mesoporous organosilica (PMO), Magnetic mesoporous organosilica drugs (MOD), Drug delivery systems, Mitoxantrone, Breast cancer treatment, Magnetic nanomedicine

## Abstract

**Background:**

Chemotherapy, the mainstay treatment for metastatic cancer, presents serious side effects due to off-target exposure. In addition to the negative impact on patients’ quality of life, side effects limit the dose that can be administered and thus the efficacy of the drug. Encapsulation of chemotherapeutic drugs in nanocarriers is a promising strategy to mitigate these issues. However, avoiding premature drug release from the nanocarriers and selectively targeting the tumour remains a challenge.

**Results:**

In this study, we present a pioneering method for drug integration into nanoparticles known as mesoporous organosilica drugs (MODs), a distinctive variant of periodic mesoporous organosilica nanoparticles (PMOs) in which the drug is an inherent component of the silica nanoparticle structure. This groundbreaking approach involves the chemical modification of drugs to produce bis-organosilane prodrugs, which act as silica precursors for MOD synthesis. Mitoxantrone (MTO), a drug used to treat metastatic breast cancer, was selected for the development of MTO@MOD nanomedicines, which demonstrated a significant reduction in breast cancer cell viability. Several MODs with different amounts of MTO were synthesised and found to be efficient nanoplatforms for the sustained delivery of MTO after biodegradation. In addition, Fe_3_O_4_ NPs were incorporated into the MODs to generate magnetic MODs to actively target the tumour and further enhance drug efficacy. Importantly, magnetic MTO@MODs underwent a Fenton reaction, which increased cancer cell death twofold compared to non-magnetic MODs.

**Conclusions:**

A new PMO-based material, MOD nanomedicines, was synthesised using the chemotherapeutic drug MTO as a silica precursor. MTO@MOD nanomedicines demonstrated their efficacy in significantly reducing the viability of breast cancer cells. In addition, we incorporated Fe_3_O_4_ into MODs to generate magnetic MODs for active tumour targeting and enhanced drug efficacy by ROS generation. These findings pave the way for the designing of silica-based multitherapeutic nanomedicines for cancer treatment with improved drug delivery, reduced side effects and enhanced efficacy.

**Supplementary Information:**

The online version contains supplementary material available at 10.1186/s12951-024-02522-4.

## Introduction

Cancer is one of the leading causes of death in developed countries, with an estimated 10 million persons calculated to have died in 2020 worldwide [1, 2]. Breast cancer in particular is the leading cause of cancer death in women with nearly 700,000 deaths in 2022 [3]. For this reason, researchers have focused their efforts on the development of diverse therapeutic strategies, although the different approaches imply different challenges. In the case of chemotherapy, non-specific distribution, lack of specificity and severe side effects cause systemic toxicity with serious implications for patients’ quality of life [4–6]. Systemic toxicity limits the dose that can be administered and thus the efficacy of the drug. Nanotechnology has emerged as a promising tool to overcome these problems [6–12] and is generating promising expectations to treat different types of cancer, such as lung cancer, breast adenocarcinoma, colon cancer, etc [10, 13, 14].

Several nanomaterials have been developed to be used as drug delivery systems (DDS), such as biodegradable polymers, hydrogels, liposomes and mesostructured silica nanocarriers [15–17]. Among them, mesoporous silica nanoparticles (MSNs) have attracted much attention because they exhibit interesting properties as drug carriers: high pore volume, uniform pore size distribution, large surface area, good chemical and thermal stability, biocompatibility, and ease of functionalization, which allows loading most of the drugs in clinical practice. In addition, these features permit the incorporation of different functionalities on the surface of the nanoparticles (NPs) to prevent drug leakage, prolong blood circulation time and allow tumour targeting. In this way, MSNs are suitable carriers to deliver the cargo to the specific target in a controlled/sustained way to achieve maximal therapeutic activity with minimal toxicity [6, 10, 13–18]. Particularly interesting are MSNs ranging from 10 to 200 nm because they preferentially accumulate in cancer cells by means of passive targeting, likely due to the enhanced permeation and retention effect (EPR effect) [19–21].

Great efforts have been made to improve the properties of inorganic MSNs by introducing organic moieties, resulting in a new generation of silica hybrid analogues: periodic mesoporous organosilica (PMO) [19, 22–32]. The introduction of organo-alkoxysilanes offers several possibilities such as: (i) higher drug loading capacity based on the ability to induce a more tuneable surface polarity able to change mesostructure-cargo interactions; (ii) improved and accurate control of cargo release through increased or decreased degradation rate of the mesostructure in biological media. Controlled release of the cargo can additionally be improved by introducing molecules sensitive to specific stimuli, which would act as gatekeepers [33–40]Thus, PMOs have emerged as a promising tool to achieve sustained and even stimuli-responsive drug release [4, 34, 35, 38, 39, 41–43].

However, the synthesis and properties of PMOs present different challenges. First, the reduction of the silanol content of the surface may reduce the colloidal stability in biological aqueous solutions. For this reason, silica precursors such as tetraethyl orthosilicate (TEOS) or bis(triethoxysilyl)ethane are frequently used as an additional source of silica. In addition, the introduction of organic bridging groups containing siloxanes, i.e. the organosilica source, may interfere with the cooperative self-assembly around the surfactant, hindering the formation of the mesoporous structure [19, 22, 26, 34, 35]. Indeed, there is only one interesting paper described in the literature on non-porous polysilsesquioxane (and therefore non-PMO) containing a pharmacologically active prodrug in the structure, based on a bis-(trialkoxy)silane derivative of platinum [40]. Therefore, the synthesis of PMOs incorporating prodrugs in their framework remains elusive and represents a major unsolved challenge that could represent a new breakthrough in the use of silica as DDS.

Mitoxantrone (MTO) is a widely used chemotherapeutic drug in the treatment of different types of leukemia and sarcoma, prostate and breast cancer [44–46]. MTO is a synthetic anthracenedione originally developed to improve the therapeutic profile of anthracyclines by avoiding their cardiotoxicity [46–48]. However, myelosuppression is an important side effect [49, 50]. MTO can readily enter into cells by passive diffusion, presumably via a “flip-flop” mechanism [46, 51], meaning that the drug can be internalized by both healthy and cancer cells [7, 20, 52]. Therefore, the delivery of MTO through NPs appears to be an attractive alternative to avoid myelosuppression but not without challenges. However, when the drug is physically adsorbed on the mesostructure of silica NPs, premature drug release may occur by diffusion. In this sense, complex gatekeepers have been used to achieve “zero premature release” [13, 16, 52].

**Fig. 1:**
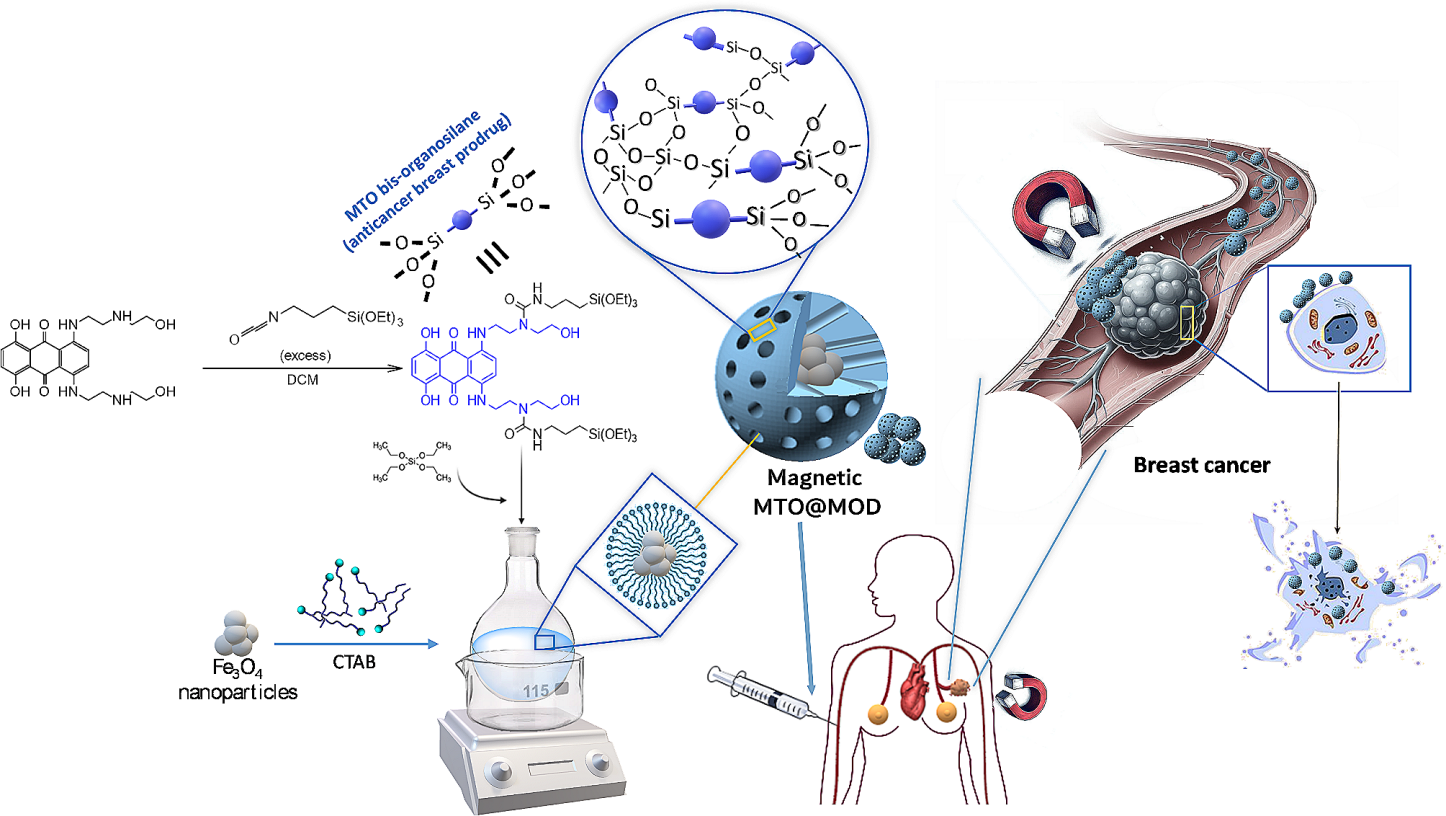
Scheme of the synthesis of MTO-bis-organosilane and MTO@MODs and treatment

In this work, a new concept of periodic mesoporous hybrid organic-inorganic silica material has been uncovered: the Mesoporous Organosilica Drugs (MODs) (Fig. 1). In this variety of PMO, the anticancer drug MTO is embedded in and forms part of the nanostructured wall, unlike conventional PMOs and MSNs in which the drugs are adsorbed in their mesoporous channels. In addition, traditional drug encapsulation requires a further step after NPs synthesis and surfactant extraction, whereas MODs combine synthesis and drug loading in a single step. To synthesize these new nanostructures, MTO has been chemically modified to obtain a bis-organosilane MTO derivative that can be used as a source of organosilica. MTO bis-organosilane plays a dual role: in addition to being a silica precursor (source of organosilica), it is a prodrug that exerts its pharmacological activity once released from the PMO framework. An important further advantage of MTO@MODs is that premature drug release is prevented, in contrast with conventional MSNs and PMOs materials, which require complex gatekeepers to avoid drug leakage [53, 54] and thus entail greater synthetic complexity and are more difficult to translate into the clinical practice. Avoidance of premature drug leakage leads to a reduction in side effects such as myelosuppression, which is characteristic of free MTO drug. Furthermore, these MOD NPs keep the drug, in its prodrug form, completely protected from the action of possible metabolites. These beneficial properties may allow lower doses of MTO to be used to achieve the same therapeutic effect, leading to a reduction in side effects [55] as well as reducing resistance. Another challenge to the conventional drug administration is their selective targeted delivery and accumulation in the organs or tissues affected. Although in the case of passive MSNs, EPR phenomenon is frequently reported, controversial results showed that the amount of gaps in the endothelial border are not so frequent to lead to tumour accumulation [56]. To overcome the drawbacks of passive MSN delivery, targeted MTO delivery was achieved in this work by means of magnetic MODs: by incorporating magnetite NPs (Fe_3_O_4_ NPs) inside MODs nanostructure they can be directed towards the desired target by using magnetic fields, thus focusing their pharmacological action on the tumour. The use of magnetic and non-magnetic MODs resulted in a significant reduction in cell viability of MCF-7 breast cancer cells for several days.

## Materials and methods

### Chemicals

Mitoxantrone (98%) was purchased from Bosche Scientific (New Brunswick, NJ, USA). Tetraethyl orthosilicate (TEOS, 98%), hexadecyltrimethylammonium bromide (CTAB ≥ 98%), dichloromethane anhydrous (DCM, ≥ 99.8%), iron (II) chloride tetrahydrate (FeCl_2_·4H_2_O, 98%), iron (III) chloride hexahydrate (FeCl_3_·6H_2_O, 97%), titanium (IV) oxysulfate solution (TiOSO_4_, 1.9 − 2.1%), hydrogen peroxide (H_2_O_2_, 32%), glacial acetic acid (≥ 99%), ammonium hydroxide solution (NH_4_OH, 30–33%), 3-(triethoxysilyl)propyl isocyanate (95%), human serum (HS), dimethyl sulfoxide (DMSO), Dulbecco’s Modified Eagle’s Medium (DMEM) (with 4,5 g L^− 1^ of glucose, stable glutamine and sodium bicarbonate), trypsin (1X), penicillin-streptomycin, L-glutamine, non-essential aminoacids, phosphate buffered saline (PBS) and Thiazolyl Blue Tetrazolium Bromide (MTT) were obtained from Sigma-Aldrich (Madrid, Spain). Absolute ethanol (EtOH), petroleum ether, sodium hydroxide and hydrochloric acid (35% w/w) were bought from Scharlab (Barcelona, Spain). Triton X-100 was purchased from PanReac AppliChem (Barcelona, Spain). DAPI (4′,6-diamidino-2-phenylindole) and Alexa Fluor 647 Conjugated Wheat Germ Agglutinin (WGA) were obtained from Fisher Scientific (Madrid, Spain).

### Synthesis of MSNs as reference material

A standard method was used for the synthesis of an MSN as reference material incorporating small variations [57]. Briefly, 160 mg of CTAB (0.44 mmol, 1 equiv.) was emulsified in Milli-Q water (68 mL) and EtOH (10 mL). The mixture was brought to pH 12 by adding NaOH (820 µL) and heated to 82 °C under magnetic stirring (500 rpm). Then, the source of silica was added (3.94 mmol of TEOS, 9 equiv.). The reaction was stopped after 2 h by cooling it with ice. The solid was recovered by filtration, washed with distilled water and EtOH and dried. To remove the surfactant, the product was redispersed in ethanolic hydrochloric acid (1 g of HCl in 100 mL of EtOH for 1 g of material) for 6 h at 60 °C. The removal of the template was checked by thermogravimetric assay and elemental analysis.

### Synthesis of the bis-organosilane mitoxantrone derivative (MTO-bis-organosilane)

The bis-organosilane precursor from mitoxantrone (MTO-bis-organosilane) was obtained through the reaction between the amino group of the aliphatic chain of MTO and the isocyanate group of 3-(triethoxysilyl)propyl isocyanate. MTO (1.5 g, 3.37 mmol, 1 equiv.) was dissolved in anhydrous DCM (50 mL) under nitrogen atmosphere. Then, the isocyanate (3.3 mL, 13.3 mmol, 4 equiv.) was dissolved in dry DCM (10 mL) and added over the previous mixture at 0 °C. The reaction was stirred at room temperature overnight. After that, the solvent was removed under reduced pressure and the solid was washed with petroleum ether to remove the unreacted isocyanate and dried under vacuum. The bis-organosilane of MTO was fully characterized by ^1^H and ^13^C NMR and MS-ESI.

### Synthesis of MODs using 100, 70, 60, 50, 20 and 10 wt% of MTO-bis-organosilane

The synthesis of MTO-100@MOD, MTO-70@MOD, MTO-60@MOD, MTO-50@MOD, MTO-20@MODs and MTO-10@MODs was performed as for the reference material (Sect. 2.2) with the addition of 100, 70, 60, 50, 20 and 10 wt% of MTO-bis-organosilane to TEOS. The molar ratio of CTAB: H_2_O: EtOH: NaOH was 1:8652:393:1.89 for MTO-20@MOD and MTO-10@MOD, and 1:6761:963:1.14 for MTO-100@MOD, MTO-70@MOD, MTO-60@MOD and MTO-50@MOD.

### Synthesis of magnetic iron nanoparticles (Fe_3_O_4_ NPs)

FeCl_3_·6H_2_O (175.5 mg, 0.65 mmol, 1 equiv.) and FeCl_2_·4H_2_O (64.5 mg, 3.24 mmol, 2 equiv.) were dissolved in Milli-Q water (9.7 mL) and glacial acetic acid (0.3 mL) at 80 ºC under mechanic stirring. After 5 min, NH_4_OH (2 mL) was added, and the reaction was stirred for 15 min. The as-made Fe_3_O_4_ NPs were washed with Milli-Q water and EtOH and dried under vacuum.

### Synthesis of magnetic MTO-20@MOD and magnetic MTO-100@MOD

Magnetic MTO-20@MODs were synthesized using the same procedure as for non-magnetic MTO-20@MOD NPs but with a difference: prior to the addition of CTAB (160 mg), 50 mg of the Fe_3_O_4_ NPs dispersed in water and sonicated for 30 min were added.

In the case of magnetic MTO-100@MODs, an additional modification was required: the CTAB content was increased to 250 mg to ensure the obtention of monodispersed NPs.

Here, the surfactant removal was accomplished by dispersing 100 mg of material in 15 mL of EtOH using ultrasounds at room temperature (3 h, 20 kHz).

### Fenton reaction with magnetic MTO-20@MOD

Fenton reaction using magnetic MTO-20@MOD was performed as follows. 180 mg of the material were dispersed in 10 mL of acetate buffer (pH = 4.5) containing H_2_O_2_ at 8 mM. The quantification of H_2_O_2_ concentration was measured through a colorimetric assay with TiOSO_4_ (1.9–2.1%).

### Characterization

^1^H NMR and ^13^C NMR spectra were obtained using a Varian Infinity 400 MHz spectrometer fitted with a 9.4 T magnetic field. ^29^Si MAS NMR experiments were performed on a Bruker Avance III/HD spectrometer equipped with a 9.4 T magnetic field. At this field strength, the ^29^Si nuclei frequency is 79.4 MHz. Samples were packed in 4 mm zirconia rotors and experiments were conducted at room temperature under Magic Angle Spinning (MAS) conditions of 12 kHz using a 4 mm H/X double-resonance MAS probe head. Chemical shift (δ) axes are shown in ppm and were externally referenced to tetramethylsilane (0 ppm). ^29^Si MAS NMR spectra were acquired by cross polarization (CP) under Hartmann-Hahn conditions with a contact time of 2ms, 2000 scans and a recycle delay of 5s. ^29^Si High Power Decoupling (HPDEC) NMR experiments were performed using a single pulse with spinal 64 decoupling (HPDEC), 1000 scans and a recycle delay of 60s. A mass spectrometer using vacuum insulated prove (VIP) heated electrospray ionization interface (Bruker UHPLC/MSMS EVOQ™ ELITE) with a triple-quadrupole detector was used for mass measurements.

The N_2_ adsorption-desorption isotherms were obtained from a Micromeritics TriStar 3000 instrument operating at − 196 ºC. Previously, the samples were treated to remove the surfactant and outgassed at 100 °C for 24 h with a N_2_ flux. The pore size distribution (PSD) was calculated using the NLDFT model for cylindrical pores.

A JEOL JEM 1400 flash microscope at 120 kV was used to characterise the morphology of the nanoparticles. The samples were first dispersed (0.04 mg mL^− 1^) in ethanol and transferred to a carbon-coated copper square mesh grids. An X-ray detector (EDS, energy dispersive spectroscopy system model TEM XPlore, from Oxford Instruments) was used for elemental mapping.

For FTIR measurements, a Mattson Infinity series instrument based on the KBr technique was used, operating in the wavelength range from 4000 to 400 cm^− 1^ with a step size of 2 cm^− 1^ and collecting 64 scans for each analysis was used. XRD analyses were collected using a Philips X’PERT MPD powder diffractometer equipped with CuKα radiation.

Thermogravimetric analyses (TGA) were performed using a Star System Mettler Thermobalance in the temperature range from 40 to 800 °C with a rate of 5 °C min^− 1^ under air atmosphere. The elemental composition was studied using a CHNS-O analyser Flash 2000 Thermo Scientific apparatus.

Hydrodynamic diameter and polydispersity index (PDI) of the samples of nanoparticles were obtained using a NanoPlus DLS Zeta potential from Micromeritics. Prior to the measurement, the samples were dispersed (0.1 mg mL^− 1^) in ultrapure water (18.2 MΩ cm) with a few drops of triton X-100 (10% in Milli-Q water).

### Drug release assay

Degradation studies were conducted by resuspending the material in human serum at a concentration of 5 mg mL^− 1^. The samples were placed in a thermo-shaker (Grant-bio) and stirring at 1400 rpm and at 37 ºC in darkness. Several samples were collected at different times and the NPs were separated from the supernatant by centrifugation at 10 000 rpm for 10 min. The solid was analysed by TEM and the MTO released was quantified by UV-vis spectrophotometry.

### Labelling of MTO-20@MODs and MTO-100@MODs with FITC

MTO-20@MODs/MTO-100@MODs (100 mg) were resuspended in dry toluene (40 mL). When the solution reached 80 °C, APTMS (2 mL) was added. The reaction was stirred at 80 °C for 24 h, and then the material was filtered, washed with toluene and EtOH and dried under vacuum.

The material functionalized with amino groups (100 mg) was dispersed in EtOH (25 mL). Then, a solution of FITC in EtOH (7.4 mg in 25 mL) was added, and the reaction was stirred at RT for 24 h in darkness. The product was filtered, washed with EtOH, dried under vacuum and stored in darkness at − 10 °C.

### Cell culture

MCF-7 human breast cancer cells (from ATCC) were cultured in DMEM supplemented with 10% foetal bovine serum and 1% penicillin-streptomycin. Cultures were grown at 37 °C in a humidified atmosphere containing 5% of CO_2_ and treated as indicated.

### Cell viability studies

Cell viability was determined by two independent methods using either flow cytometry or colorimetry.

For colorimetric estimation of cell viability, cells were seeded in 12-well plates at a density of 12 000 cells per well and treated with the different materials and controls for 3 and 7 days after incubation for 24 h. Then, 3-(4,5-dimethylthiazol-2-yl)-2,5-diphenyltetrazolium bromide (MTT) was added at a final concentration of 0.1 mg mL^− 1^. In living cells with an active metabolism, the MTT is reduced to formazan, a purple product [58]. After incubating the cells for 3 h at 37 °C, the medium was removed and DMSO was added, so the cells were permeabilized. Once dissolved in DMSO, the formazan was transferred to a 96-well plate and the absorbance was measured at 542 nm using a Spectra FLUOR (Tecan).

For flow cytometry, MCF7 cells treated with the different materials for 1, 3 or 7 days were washed with PBS solution and stained 10 min in the dark at 37 °C with propidium iodide (PI, Sigma-Aldrich), a DNA intercalating dyes excluded from viable cells due to membrane impermeability. Only apoptotic and necrotic cells are stained by these dyes. The PI was excited at 305 nm and analysed at 615 nm. The number of cells stained was analysed using a CXP software. The percentages of cells stained or not with dyes were analysed using CXP software (Becton Dickinson). For each treatment, measurements were conducted in at least three independent experiments, and, in each experiment every condition was assessed by triplicated.

### Cellular uptake

Cells were seeded at a density of 50 000 cells per well in 6-well plates and treated with the selected FITC-labelled materials carrying MTO after 24 h. MTO and MTO-bis-silane were used as positive controls and cells treated with the reference material or untreated were used as negative controls. At different times (1, 3 and 7 days) cells were washed twice, resuspended in PBS solution, and analysed by flow cytometry (FACSCalibur, Becton Dickinson). Intracellular FITC-labelled materials were excited at 488 nm and analysed at 520 nm using CXP software (Becton Dickinson). Each treatment was administrated by duplicated and the experiment was repeated three times.

### Confocal imaging

The MCF-7 cells were grown on cover slips and treated with the selected FITC-labelled NPs. After washing with PBS, the cells were stained with fluorescent WGA (1 µg mL^− 1^) and DAPI (1:10000) to reveal cell membranes and nucleus, respectively. Images were acquired using a FV3000 confocal microscope (Olympus) with a 63x objective.

## Results and discussion

### Synthesis and characterization of the bis-organosilane derivative of Mitoxantrone (MTO-bis-organosilane)

The aim of this work is the synthesis of a new type of PMO, Mesoporous Organosilica Drugs (MODs), made up of the chemotherapeutic drug MTO. Importantly, the novelty of these new MODs NPs lies in the incorporation of the drug in the form of a prodrug that is part of the 3D framework of the NPs, unlike conventional MSNs or PMOs, in which the drugs are adsorbed into the inner porosity or covalently grafted to the external surface silanol groups of these NPs. Therefore, the first objective in the design of MODs was to synthesize a bis-organosilane derivative of the drug MTO that could be used as a bridged silica precursor for the synthesis of MODs. To do this, the molecule must carry alkoxysilane groups that can participate in the condensation reaction that builds the silica framework. In this case, we based our approach on the synthesis of a bis-organosilane with two triethoxysilane groups attached to mitoxantrone through a urea linkage. These groups were introduced by reacting the amino groups of the aliphatic chain of MTO with the isocyanate group from 3-(triethoxysilyl)propyl isocyanate, using DCM as a solvent (See Fig. 1). The reaction was performed by using an excess of the isocyanate to ensure complete conversion of the starting MTO. The excess of this reagent could be removed by washing the crude product several times with petroleum ether.

The successful formation of the bis-organosilane MTO derivative was confirmed by ^1^H and ^13^C NMR spectroscopy. The new molecule shows a new signal corresponding to the proton of the –*NH* group of the amide bond, which appears as a triplet at 6.01 ppm (see Figure S1 in the Supporting Information). The presence of the signals of the protons of the –C*H*_*2*_C*H*_*3*_ groups of the triethoxisilane groups and their adequate integration confirmed that the product remains non-hydrolysed. The NMR spectra showed no impurities. The formation of the desired bis-organosilane can also be confirmed by the signal of the newly formed amide carbon in the ^13^C NMR spectrum at 159.9 ppm (see Figure S1). The MTO-bis-organosilane derivative was also characterized using FTIR measurements (see Figure S2). The spectrum of the bis-organosilane shows the stretching vibration bands at 1080 cm^− 1^, corresponding to the presence of Si-O bonds in the molecule, and the C = O bond from the amide group at 1562 cm^− 1^, confirming the formation of the desired MTO bis-organosilane molecule. In addition, mass spectrometry in the positive method was performed to determine the exact mass of the molecule, resulting in the value M + Na^+^ 961.4 (See Figure S3).

### Synthesis and characterization of mitoxantrone-modified mesoporous organosilica drug nanoparticles (MTO@MODs)

Having successfully synthesised the silica precursor based on the modification of the MTO drug, the MTO bis-organosilane molecule, the next step is to synthesise a new type of PMO, the mesoporous organosilica drugs (MODs), with the aim of overcoming one of the challenges of drug delivery systems: avoiding drug leakage from the carriers before the NPs reach their target and protecting the drugs from enzyme or metabolite attack. By incorporating the MTO drug into the MOD mesostructure, as a bis-organosilane MTO prodrug, we expected that premature drug release would be remarkably lower or negligible compared to the case of conventional procedures in which the drug is physisorbed on the nanocarrier, as the MODs need to be degraded or semi-degraded to release the drug. In addition, since carrier uptake by cancer cells is enhanced for nanometre-sized NPs, we aim to synthesize MODs in the range of 10 to 200 nm. For this purpose, we modified a standard procedure [57] for MSNs synthesis: the variation introduced was the addition of the MTO-bis-organosilane (dissolved in EtOH) together with TEOS as silica source (Fig. 1).

MTO@MODs with different amounts of the MTO-bis-silane prodrug were synthesized and characterized. Conventional MSNs were also synthesised as reference material. Initially, the challenge of synthesising a purely organic MOD (MTO-100@MOD) using the bis-organosilane derivative of MTO as the only source of silica was investigated. To this end, an optimisation of the procedure for the synthesis of the reference MSNs was carried out (see SI for a detailed discussion). The optimization process consisted of: (1) isolating and purifying the MTO-bis-organosilane, (2) reducing the amount of MTO-bis-organosilane added to the synthesis media to decrease the particle diameter, (3) increasing the ethanol content relative to water and (4) decreasing the amount of base to reduce the polymerization rate. The specific synthesis conditions are summarized in Table S1, and the different attempts performed can be observed in Figure S5.

**Fig. 2:**
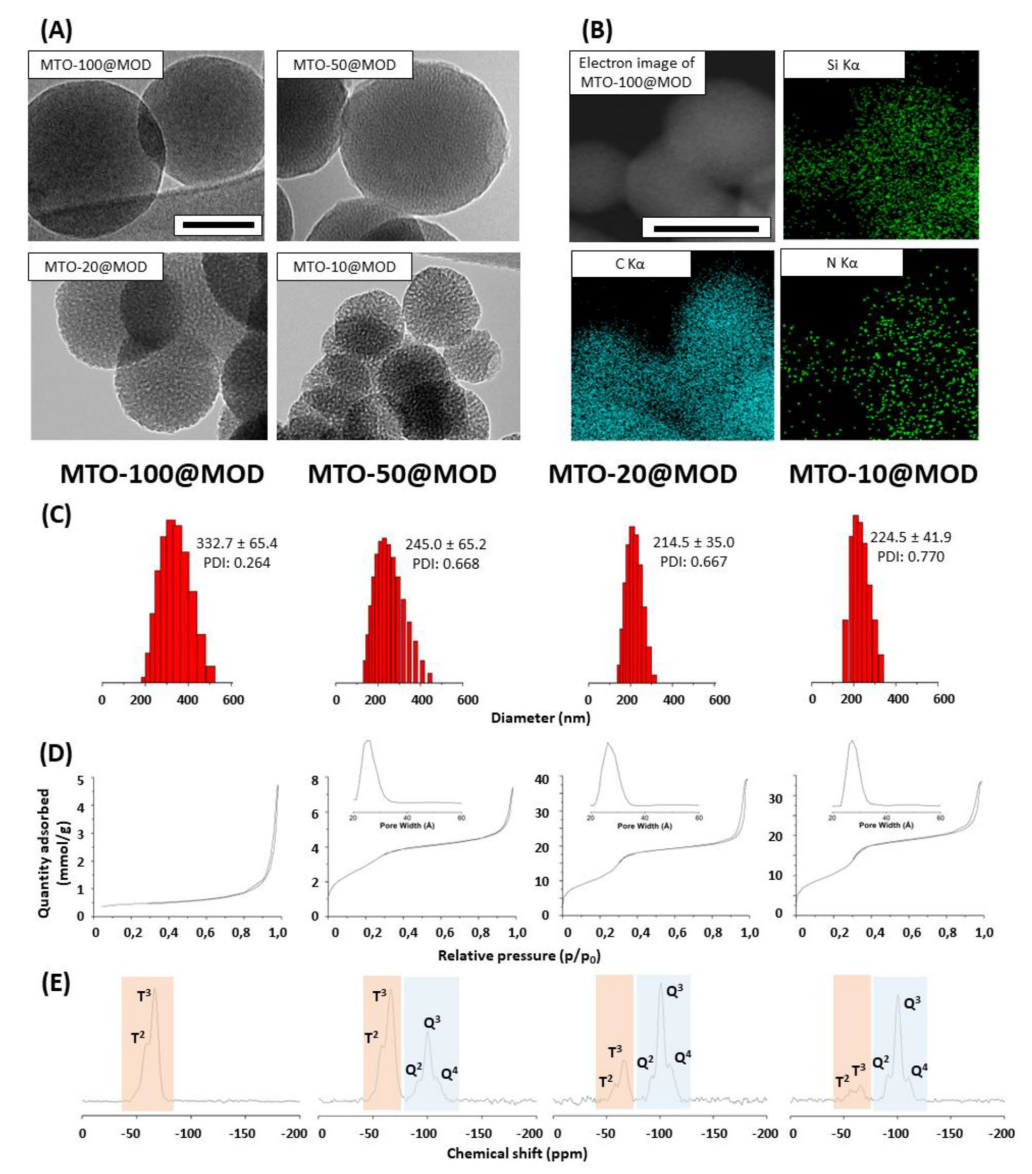
TEM images of MTO-100@MOD, MTO-50@MOD, MTO-20@MOD and MTO-10@MOD **(A)**; mapping (of MTO-100@MOD) **(B)**; hydrodynamic diameter measured by DLS **(C)**; N_2_ adsorption-desorption isotherm and PSDs (when proceed) based on NLDFT model **(D)**; and CP/MAS ^29^Si solid-state NMR spectra from left to right of MTO-100@MOD, MTO-50@MOD, MTO-20@MOD and MTO-10@MOD(E). Scale bars represent 50 nm

The obtained MOD NPs were spherical, monodisperse and nanometre sized, as shown by TEM images (Fig. 2A; Table 1) and DLS measurement (Fig. 2C). The organic nature of MTO-100@MOD was confirmed by TEM microanalysis as the presence of C atoms was homogeneous throughout the whole sample (Fig. 2B). Thermogravimetric analysis revealed that, as expected (Table S2), only a 21 wt% of the sample remained after exposure to heat and air (Figure S6), corresponding to the inorganic silicon moieties. The elemental analysis is displayed in Table S3, and the results also shows that the expected theoretical values for the % of C and N in the MTO-100@MOD and the obtained experimental values are in good agreement. However, the N_2_ adsorption-desorption isotherm is of type II according to the IUPAC classification, which is indicative of non-porous materials (Fig. 2D), consistent with a BET surface area corresponding roughly to that of a dense NP (32 m^2^ g^− 1^) [57]. However, the role of the surfactant was found to be essential for the ordering and spherical geometry of this dense MOD nanostructure. The synthesis of the MOD material has been carried out in the absence of surfactant, obtaining dense nanostructures but without any ordering in morphology and geometry (Figure S7).

It can be concluded that the use of pure bis-organoalkoxysilanes as a silica source can prevent the formation of a mesoporous structure [19, 34, 35]. For this reason, we hypothesized that the introduction of an inorganic silica source such as tetraethoxysilane (TEOS) would improve the textural properties of the materials. We then performed several syntheses with different ratios of MTO-bis-silane/TEOS (Table 1). In the first attempt, the amount of MTO-bis-silane added in weight was set at 70 wt% (MTO-70@MOD), with the remaining 30 wt% being TEOS. The reaction conditions for the synthesis were those optimized for MTO-100@MOD. The size and morphology of the obtained material were similar to those of MTO-100@MOD. MTO-70@MOD also showed very poor textural properties with a BET surface area of 41 m^2^ g^− 1^ and a total pore volume of 0.06 cm^3^ g^− 1^ (Table 1, entry 2). When the MTO-bis-silane content was reduced to 60 wt% (MTO-60@MOD), a slight increase in the BET surface area to 92 m^2^ g^− 1^ was observed (Table 1, entry 3).

**Table 1:** Textural properties and diameter of different MODs varying the % in weight of MTO-bis-organosilane and TEOS

Entry	Material	BET area (m^2^ g^− 1^)	Pore volume (cm^3^ g^− 1^)	PSD (nm)^1^	Diameter nm)^2^
1	MTO-100@MOD	32	0.05	n.d.	107
2	MTO-70@MOD	41	0.06	n.d.	96
3	MTO-60@MOD	92	0.09	n.d.	96
4	MTO-50@MOD	241	0.26	2.6 (2.1–3.1)	92
5	MTO-20@MOD	950	0.93	2.7 (2.2–3.4)	49
6	MTO-10@MOD	896	0.95	2.7 (2.4–3.4)	50
7	Magnetic MTO-20@MOD	614	0.30	3.15 (2.8–3.8)	158
8	Magnetic MTO-100@MOD	32	0.04	n.d.	179

^1^ PSD based on NLDFT model. Maximum of the distribution followed by PSD in parentheses. ^2^ Measured using TEM micrographs. n.d. = not determined

Increasing the amount of TEOS to 50 wt% (MTO-50@MOD) resulted in a more significant improvement in the textural properties of the sample (Table 1, entry 4). The N_2_ adsorption-desorption isotherm is of type IV, and the BET surface area increased to 241 m^2^ g^− 1^ with a total pore volume of 0.26 cm^3^ g^− 1^ (Fig. 2D). Furthermore, the pore size distribution reveals mesopores with a maximum around 2.6 nm. In addition, the XRD pattern shows a prominent X-ray reflection peak in the range of 1.5-3 (2θ), indicating the presence of mesostructure (Figure S8). Finally, the mesoporous framework can also be observed in the TEM images (Fig. 2A). The materials were also characterized by DLS (Fig. 2C) and thermogravimetric analysis and showed a decreased of mass of 56% (Figure S6), as expected. Thus, MTO-50@MOD is the first PMO with remarkable textural properties synthesized with an anticancer drug in the silica framework and accessible internal porosity.

Next, to widen the range we decided to synthesize, characterize and compare materials with a lower proportion of MTO-bis-organosilane. The attempts included 20 and 10% by weight of added bis-organosilane (MTO-20@MOD and MTO-10@MOD). The nitrogen adsorption-desorption isotherms are of type IV according to the IUPAC classification, indicating the capillary condensation step at a relative pressure of 0.25–0.3 and a significant filling of mesopores (Fig. 2D). Both materials exhibited large BET surface areas of around 900 m^2^g^− 1^ and pore volumes of around 1 cm^3^g^− 1^ (Table 1, entries 5 and 6), like the MSNs reference material (Figure S4). The XRD spectra also showed the corresponding typical pattern for mesoporous frameworks (Figure S8). Additionally, the NP size of MODs measuredusing TEM micrographs (Fig. 2A) and showed dispersed NPs with diameters around 50 nm (Table 1, entries 5 and 6). DLS measurement (Fig. 2C) confirmed the homogeneous size distribution and showed a larger diameter, as expected.

The synthesized MODs were also characterized using CP/MAS ^29^Si solid-state NMR (Fig. 2E). The material synthesized with MTO-bis-organosilane as the only silica source (MTO-100@MOD) shows a single band with two signals at − 59 ppm and − 69 ppm which are assigned to T^2^ and T^3^ sites, respectively. Interestingly, the T^3^ sites are the predominant ones in the sample suggesting that most of the triethoxysilane groups of the MTO-bis-silane have been converted to siloxane groups during the polymerization process. In the case of the MOD NPs synthesized with 50% of MTO-bis-silane, two different regions are observed: the first one is the result of the condensation of the bis-silane with the same two signals as MTO-100@MOD materials and assigned to the T^2^ and T^3^ sites; while the other corresponds to signals at − 92, − 100 and − 108 ppm assigned to Q^2^, Q^3^ and Q^4 29^Si species, respectively, from the condensation of TEOS. The intensity of the Q and T sites is related to the amount of inorganic and organic silica content. For MTO-20@MOD, the intensity of the Q band is increased with respect to MTO-50@MOD, while the T band decreases, as it can be observed from the data of the different areas (Table S4). The same tendency is observed in the case of MTO-10@MOD. Then, as the amount of MTO-bis-silane moiety decreases, the intensity of the band corresponding to the T sites decreases. Overall, it can be concluded that as the ratio of TEOS in these MODs increases, the intensity of the Q sites relative to the T sites in the material also increases.

Then, since the synthesis of MTO@MODs was successfully accomplished, thus obtaining a new type of PMO with a prodrug as part of its 3D framework.

### Release experiments of mitoxantrone-modified mesoporous organosilica drug nanoparticles (MTO@MODs)

**Fig. 3:**
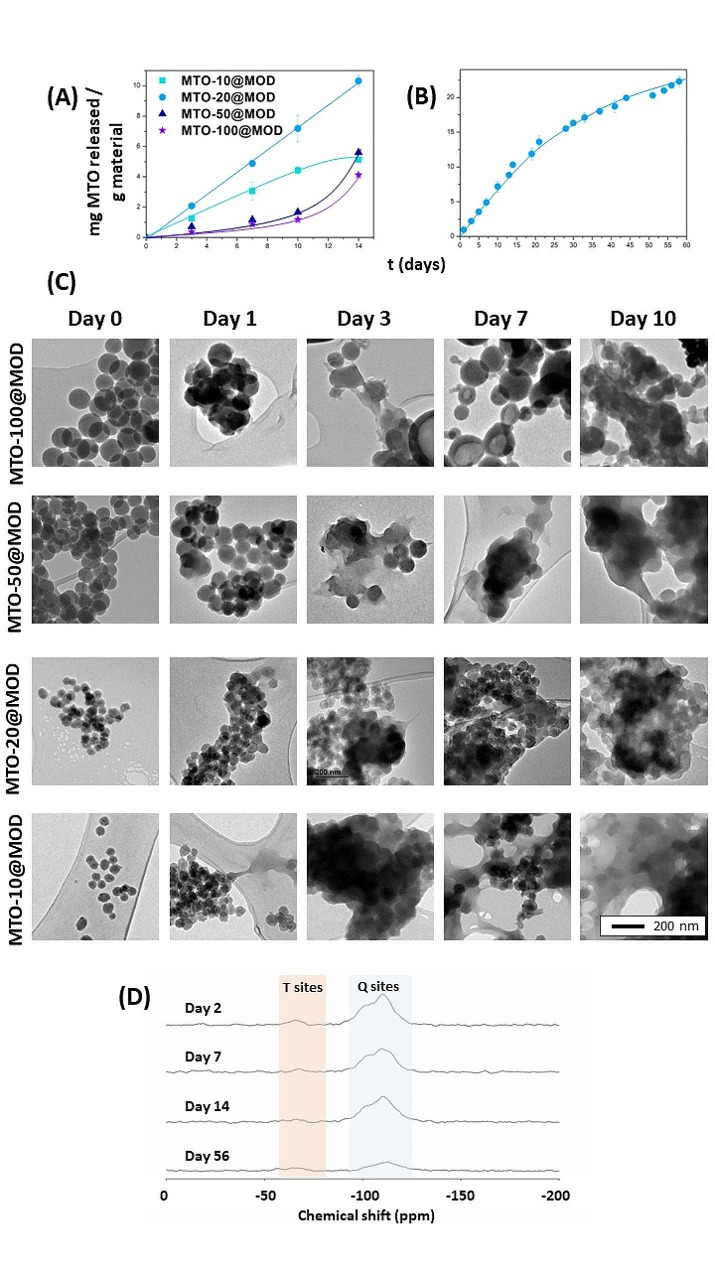
**(A-B)** Quantification of the amount of MTO released by UV-Vis spectrometry (λ = 620 nm). **(A)** Time course of release and degradation of MTO-100@MOD, MTO-50@MOD, MTO-20@MOD and MTO-10@MOD exposed to human serum at 37 °C and 1400 rpm. **(B)** Detailed release of MTO from MTO-20@MOD for longer times. **(C)** TEM images of materials biodegraded by human serum on different days. **(D)** ^29^Si HPDEC/MAS solid-state NMR spectra of MTO-20@MOD upon degradation by human serum during 2, 7, 14 and 56 days highlighting T and Q sites

As a result of the incorporation of the MTO-silane prodrug into the three-dimensional structure of the MODs, biodegradation of the MODs will be necessary for sustained drug release. To investigate whether the materials would be degraded in normal tissue or in tumour microenvironment and, subsequently, where the MTO prodrug would be released from its matrix, we performed different release assays. The experiments consisted of exposing the materials to an environment that simulated the physiological pH conditions in the human body and the tumour microenvironment. Briefly, the materials were stirred at a concentration of 5 mg mL^− 1^ at 37 °C in two different buffers, PBS at pH = 7.4 to simulate physiological pH and acetate buffer at pH = 4.5 to simulate lysosomal conditions, which are even harsher than tumour microenvironment (reported to be between 5.6 and 6.8) [59]. A sample of each material was removed over time. After centrifugation, the absorbance of the supernatants was measured using a UV-Vis spectrometer to determine the quantity of MTO released. Neither MTO-bis-organosilane nor MTO could be detected in any of these experiments (data not shown) indicating that MODs are not degraded under these conditions. Then, the release experiment was conducted using a medium more similar to the blood: human serum. In this case, a sustained drug release over time was observed for all materials probably due to enzymatic activity (Fig. 3A and B). TEM monitoring revealed morphological changes during the degradation process (Fig. 3C) including deformation, merge, and decomposition of the NPs boundaries as well as debris of NPs. It is noteworthy that the process was accelerated as the inner porosity and surface area increased from MTO-100@MOD to MTO-10@MOD. In addition, long-term biodegradation experiments resulted in the complete loss of mesoporosity and full disordering of the framework (Figure S9).

The highest release rate of MTO per g of NPs was obtained for the material MTO-20@MOD, while MTO-100@MOD and MTO-50@MOD exhibited the lowest release rate (Fig. 3A), despite carrying the highest amount of drug in their framework. This may be a consequence of the textural properties of the different materials. MTO-20@MOD has the highest surface area due to its higher inner porosity, which is much larger than the surface area of MTO-50@MODs and MTO-100@MODs. Thus, the area exposed to degradation by the media is remarkably larger in MTO-20@MOD, resulting in a faster degradation and therefore a faster release of the prodrug from the host to the media. The difference in the amount of MTO released between MTO-10@MOD and MTO-20@MOD, which have a fairly similar surface area, may be a consequence of the different amount of MTO in each material.

Drug release experiments of conventionally encapsulated drugs are usually performed in aqueous buffer and generally result in a burst release of the drug. In contrast, here a much more degradative medium (human serum contains enzymes) was used to release the drug from the NPs. Continuous and sustained drug release for 14 days was observed for MTO-10 and MTO-20@MOD. Encouraged by these results, the release of MTO from the MTO-20@MOD was followed over two months, to confirm continuous and sustained drug release over time, with no clear tendency to reach a plateau, indicative of release completion or equilibrium (Fig. 3B). In addition, ^29^Si-RMN was used to analyse the samples of MTO-20@MOD exposed to human serum degradation for different periods of time (Fig. 3D). Interestingly, a significant reduction in the number of Q and T sites was observed over time, suggesting that the degradation of the MODs occurs mainly by hydrolysis of the siloxane groups, which are further converted to silanols and finally lead to leaching of the different silica species [60]. Thus, the cleavage of the Si-O-Si bonds triggers the release of MTO as a prodrug in a continuous and sustained manner, in good agreement with the drug release results observed in human serum.

The percentage of MTO prodrug released from the materials MTO-50@MOD and MTO-100@MOD is practically negligible until day 10 (Fig. 3A). These materials, synthesised with a higher amount of MTO bis-organosilane relative to TEOS, have a lower surface area than MTO-10@MOD and MTO-20@MOD, as well as a lower porosity, which results in a much smaller surface area exposed to the medium to undergo biodegradation. In conclusion, the adjustable rate of drug release is a remarkable feature of MOD nanoparticles. MODs can be designed to tune the rate of drug release by changing the amount of prodrug introduced into their framework, thus adapting to the sensitivity of the patient and the degree of disease progression.

### Biological in vitro evaluation of mitoxantrone-modified mesoporous organosilica drug nanoparticles (MTO@MODs) in breast cancer cells viability

As MTO is a treatment for breast cancer, the biological effect of the MTO@MOD materials was investigated in vitro to assess their cytotoxicity on a well characterised breast cancer cell line, MCF-7. The slow release of MTO exhibited by MODs should lead to a parallel slow decline in cell viability. For this reason, MTT assays were designed to follow cell viability effects of MTO@MODs at 3 and 7 days. As proper controls, MTO alone was added to the culture medium of the cells. Besides, in order to guarantee the cytotoxic activity of the MOD NPs, it was first necessary to prove that the MTO-bis-organosilane itself had cytotoxic capacity. For this purpose, several solutions of MTO-bis-organosilane were also prepared in DMEM at different concentrations and used to treat MCF-7 cells (Figure S10). As expected, MTO-bis-organosilane causes breast cancer cell death although at higher concentration than free MTO drug. Therefore, the observed cytotoxic activity of MTO-bis-organosilane ensures that MTO@MODs have the ability to effectively reduce the viability of breast cancer cells. Thus, following the cell viability decrease during the experiment time, doses of 0.15 µM for MTO and 25 µM for MTO-bis-organosilane were chosen as positive controls.

Moreover, the biocompatibility of the pure silica host (reference MSN) was assessed and found no change in the cell viability for cells treated with the reference materials as compared to the negative control without treatment (Figure S11). The good biocompatibility of these pure silica materials has been previously reported [18, 34, 35].

**Fig. 4:**
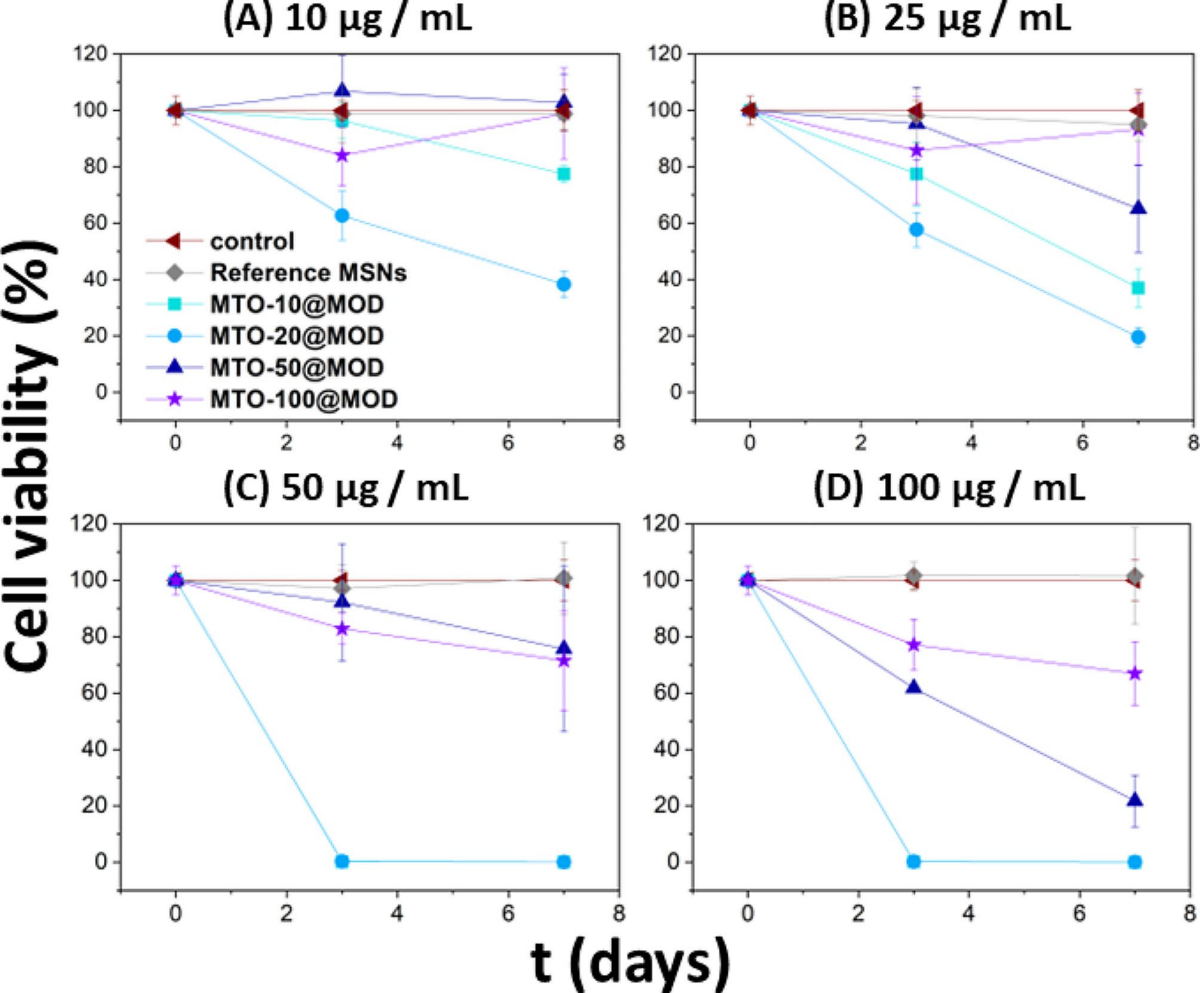
Cell viability loss of MCF-7 breast cancer cells using MTT assay after treatment with MTO-10@MODs, MTO-20@MODs, MTO- 50@MODs and MTO-100@MODs at a dose of 10 µg mL^− 1^ **(A)**, 25 µg mL^− 1^ **(B)**, 50 µg mL^− 1^ **(C)** and 100 µg mL^− 1^ **(D)**

The effect of MTO@MOD NPs was investigated using different doses. At a dose of 10 µg mL^− 1^ (Fig. 4A), MTO-20@MOD is the only material that induces a significant decrease in cell viability in a week. At increased doses of 25 µg mL^− 1^ MTO-10@MOD NP also reduce cell viability (Fig. 4B). However, MTO-50@MOD and MTO-100@MOD caused no significant changes in cell viability at these doses, despite carrying a remarkably higher amount of MTO. A plausible explanation may be found in the different textural properties of the materials. The nanoparticles with higher surface area (MTO-20@MOD and MTO-10@MOD) have a higher rate of degradation and therefore a higher rate of drug release and thus, elicit a higher decrease in cell viability, even though the MODs with lower surface area carry a higher amount of drug (MTO-50@MOD and MTO-100@MOD). Faster degradation of materials with higher BET areas was already demonstrated in the release assay. The different behaviour of MTO-20@MOD and MTO-10@MOD may be explained by the different amount of MTO incorporated in their structure. From these results, it can be concluded that MTO-20@MOD exhibits the most suitable behaviour, as a significant decrease in the cell viability can be observed at the lowest dose used.

Higher doses (50 and 100 µg mL^− 1^) were used to further investigate the cytotoxic behaviour of the MODs. At both doses, MTO-10@MOD and MTO-20@MOD reduced cell viability by 100% in a short time half week (Fig. 4C and D). As for MTO-50@MOD and MTO-100@MOD samples, there are no significant differences between the two materials at 50 µg mL^− 1^, although some cytotoxic activity (around 30%) is observed after 7 days. Interestingly, an increased dose of 100 µg mL^− 1^ exerted differential cytotoxicity, with MTO-50@MOD reducing by 80% cell viability after 7 days, whereas MTO-100@MOD reduced cell viability only by 30%, which was an effect similar to that observed with 50 µg mL^− 1^. Again, the most likely explanation for this result is that the material with higher porosity and specific surface area undergoes greater biodegradation, allowing a faster release of the pharmacologically active species, mainly the MTO prodrug.

It should be noted that MOD NPs have a sustained release profile over time, even in long-term in vitro cell viability studies of more than 7 days, where the growth rate of tumour cells in a favourable medium is usually higher than the cytotoxicity rate. The fact that MODs reduce the viability of breast cancer cells for several days while preventing premature release of active species, as demonstrated, is a promising result for reducing chemotherapy-related side effects.

**Fig. 5:**
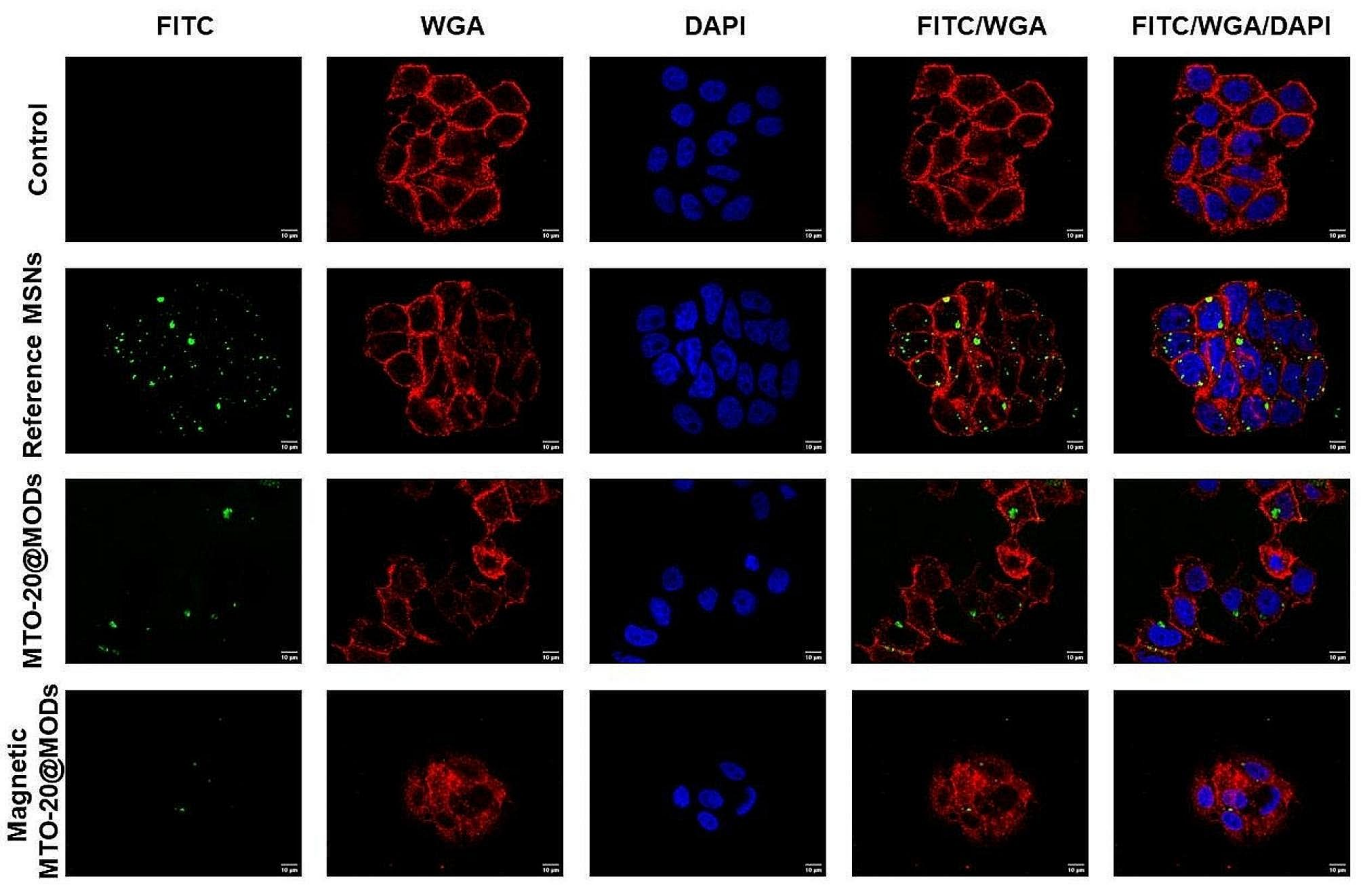
Confocal imaging (single plane) of MCF-7 cells incubated with 25 µg mL^− 1^ of FITC-conjugated reference MSNs and MTO-20@MODs. Control cells were cultured without NPs. Reference MSNs and MTO-20@MODs were conjugated with FITC (green), cell membranes were stained with WGA (red) and nuclei with DAPI (blue)

Finally, MTO-20@MODs cellular internalization was evaluated by confocal imaging at the equatorial plane of the cells (Fig. 5). The generated image corresponds to a specific depth within the sample, thus ensuring colocalization of the captured objects. MTO-20@MODs was labelled by conjugation with the fluorochrome FITC (green), cell membranes were stained with WGA (red) and nuclei were revealed with DAPI (blue). Efficient cellular internalization of MTO-20@MODs is appreciated in the merged pictures of bottom panels (FITC/WGA or FITC/WGA/DAPI). Untreated cells served as negative controls and cells treated with reference NPs labelled with FITC but not containing MTO served as positive internalization controls. The dose of reference NPs and MTO-20@MODs used was 25 µg mL^− 1^. MTO and MTO-bis-organosilane showed no fluorescence in the FITC wavelength (Figure S12), as expected.

In line with the confocal images, quantification of cellular uptake using flow cytometry revealed higher internalization rate for the reference MSNs than for MTO-20@MODs (Figure S13). The high cytotoxicity of MTO-20@MODs may be responsible of the fewer living cells with these NPs inside. To explore this possibility, we evaluated cell viability reductions in MCF-7 cells treated with MTO-20@MOD NPs by flow cytometry (Figure S14). Indeed, cell viability reductions of more than 50% by day 3 and almost 90% by day 7, in line with previous MTT assays, support this idea.

### Synthesis and characterization of magnetic mitoxantrone-modified mesoporous organosilica drug nanoparticles (magnetic MTO@MODs)

Several studies have questioned the enhanced permeability and retention (EPR) effect as the paradigm for retention and passive accumulation of nanomedicines in solid tumours, highlighting the importance of active targeting therapies [6, 61, 62]. For this reason, we aim to direct MTO-20@MOD and the purely organic MTO-100@MOD, to the desired target. We then re-engineered the previously obtained MOD nanomedicines by introducing magnetic NPs into their structure for magnetic targeting (Fig. 1). The synthetic strategy consisted of dispersing magnetic Fe_3_O_4_ NPs by ultrasonication before adding the CTAB surfactant, so that the magnetic NPs are surrounded by the surfactant molecules inside the micelles. Magnetic Fe_3_O_4_ NPs were prepared by co-precipitation using iron chloride salts as precursors, and ammonium hydroxide as catalyst.

**Fig. 6:**
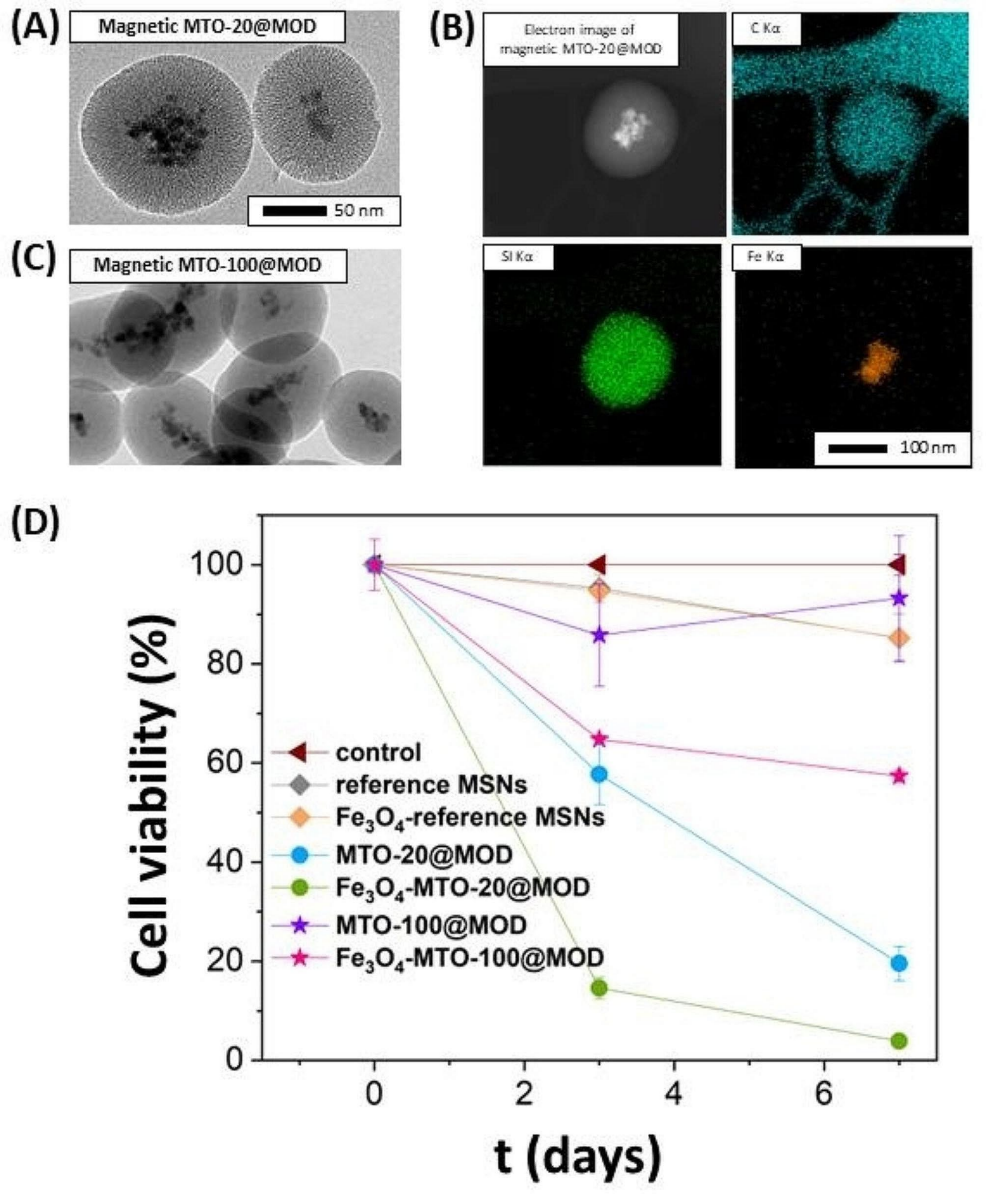
TEM micrograph **(A)** and mapping **(B)** of magnetic MTO-20@MODs. TEM micrograph of magnetic MTO-100@MOD **(C)**. Effect over MCF-7 cancer cells viability of magnetic and non-magnetic reference MSNs, MTO-20@MODs and MTO-100@MODs at 25 µg mL^− 1^ **(D)**

The magnetic MTO-20@MODs retained their spherical shape, and slightly increased their size as shown by TEM micrographs (Fig. 6A). The incorporation of the Fe_3_O_4_ NPs was confirmed by EDS elemental mapping (Fig. 6B) and quantified from the TEM images. Once synthesized, up to 68% of the NPs were magnetic. We increased the proportion to 98% by separating the magnetic MODs from those without magnetite by using a magnet. The good dispersion of the NPs and textural properties were also retained as shown by the N_2_ isotherms (Figure S15) and the DLS measurement (Figure S16).

Interestingly, magnetic MTO-20@MOD exerted a significantly higher reduction in breast cancer cells viability than their non-magnetic counterparts at 25 µg mL^− 1^ (Fig. 6D). As a control, the reference magnetic MSNs had no effect on cell viability. Therefore, the enhanced cytotoxic effect of magnetic MTO-20@MOD may be a consequence of a synergistic effect between magnetite and MTO. Previously, other authors have proved that magnetite catalyses the Fenton reaction, in which the decomposition of hydrogen peroxide (H_2_O_2_) produces toxic free radicals (reactive oxygen species, ROS) [63–65]. High concentration of H_2_O_2_ has been recognized as a hallmark of the tumour microenvironment [66, 67]. In addition, MTO can be oxidized by ROS to produce different metabolites, which have been reported to be the active ingredient of the drug [68–71]. Magnetic MODs could then catalyse the conversion of H_2_O_2_ into free radicals, triggering MOD degradation, MTO release and oxidation. Toxic free radicals together with oxidized MTO metabolites would then have a synergistic effect promoting cancer cell death. To prove this hypothesis, magnetic MTO-20@MODs were dispersed in aqueous solution containing H_2_O_2_. Quantification of H_2_O_2_ content over time (Figure S17) demonstrated a reduction that supports that magnetic MTO-20@MODs catalyse the Fenton reaction. The efficient internalization of the magnetic MTO-20@MODs was also confirmed by confocal imaging (Fig. 5).

Encouraged by these results, we aimed to investigate whether if magnetite introduction could trigger the degradation of MTO-100@MOD and thus enhance its cytotoxicity. Magnetic MTO-100@MOD retained the spherical shape and were larger than MTO-100@MOD (Fig. 6C and S18). Indeed, the MTT assay demonstrated that magnetic MTO-100@MOD had significantly higher cytotoxicity, more than 20%, compared to its non-magnetic counterpart.

In conclusion, newly synthesized magnetic MTO-20@MOD and magnetic MTO-100@MOD have shown a great potential as DDSs to specifically deliver the chemotherapeutic drug MTO to the tumour by using magnetic fields with tissue penetration ability. Furthermore, the introduction of magnetite triggers the degradation of these materials through the generation of ROS by the Fenton reaction. This releases MTO-bis-organosilane prodrug, which together with ROS species and local magnetic hyperthermia, also induced by magnetite NPs, would synergistically enhance the cytotoxicity of these nanocarriers, thus achieving a three-in-one trimodal therapeutic nanomedicine.

## Conclusions

In conclusion, we have successfully synthesized a bis-organosilane derivative of the chemotherapeutic drug mitoxantrone to be used as a prodrug silica source for the synthesis of a novel type of PMOs: Mesoporous Organosilica Drug (MODs), which differ from conventional PMOs by carrying the drug covalently embedded in the NP framework and potentially capable to carry additional synergistic drugs. Several MODs with different content of mitoxantrone prodrug (MTO bis-organosilane derivative) have been synthesised even with 100% purely organic bridged material. These MODs exhibit properties suitable for use in nanomedicine: the optimized materials were spherical, nanometre-sized, and with relative mesoporosity depending on the incorporation of MTO bis-organosilane in the framework.

Interestingly, the nanomaterials MTO-10@MOD and MTO-20@MOD showed higher and faster drug release rates than MTO-50@MOD and MTO-100@MOD, despite their lower drug content. This fact, which may be explained because the surface area exposed to environmental degradation is greater, is associated with a greater reduction in cell viability in MCF-7 breast cancer cells. The higher release rate and efficacy in reducing cell viability was observed for the MTO-20@MOD material. However, depending on the specificity of the patients and their required treatment conditions, the material obtained from the pure MTO-bis-organosilane prodrug, MTO-100@MOD, could be used for slow and prolonged treatment, and therefore the adjustable drug release rate is a remarkable feature of MOD nanoparticles.

Importantly, by incorporating the drug into the NPs framework, we achieved the goal of avoiding premature drug release, unlike traditional MSNs and PMOs that carry the drug adsorbed in their channels. Subsequent MODs biodegradation enabled MTO prodrug sustained release at the target cancer tissue, thereby minimising the side effects. In addition, Fe_3_O_4_ NPs were incorporated into the MODs to obtain magnetic MOD NPs, with the aim of active targeting to direct the MODs to the tumour site by means of alternating magnetic fields, thereby increasing the efficacy of the drug. In addition, magnetic MODs facilitate the Fenton reaction by reacting with hydrogen peroxide that exist in cancer environment, promoting the generation of ROS capable of killing cancer cells. This cooperates with and enhances the efficacy of the MTO drug loaded into the NPs. Cancer cell viability was greatly reduced by magnetic MTO@MODs, and cytotoxicity was more than doubled after 3 days compared to non-magnetic MTO@MODs. Magnetic MODs are therefore promising materials that could significantly increase the efficacy of a given chemotherapy, while avoiding side effects and reducing the toxicity of free circulating drugs in the body, such as MTO. New promising insights are the synthesis of MODs carrying two or more drugs to benefit from combination therapy to achieve synergistic effects.

### Electronic supplementary material

Below is the link to the electronic supplementary material.

Supplementary Material 1

## Data Availability

No datasets were generated or analysed during the current study.
