# Supplementary material for: Magnetically modified-mitoxantrone mesoporous organosilica drugs: an emergent multimodal nanochemotherapy for breast cancer
Source: J Nanobiotechnology. 2024 May 14;22:249. doi: 10.1186/s12951-024-02522-4 (PMC11092073; doi:10.1186/s12951-024-02522-4)
Supplement: Supplementary file 1 — Supplementary Material 1 [file 12951_2024_2522_MOESM1_ESM.docx]

**Supporting Information**

**Characterization of the bis-organosilane of mitoxantrone (MTO-bis-organosilane)**

**1,1'-(((5,8-dihydroxy-9,10-dioxo-9,10-dihydroanthracene-1,4- diyl)bis(azanediyl))bis**

**(ethane-2,1-diyl))bis(1-(2-hydroxyethyl)-3-(3-(triethoxysilyl)propyl)urea)**


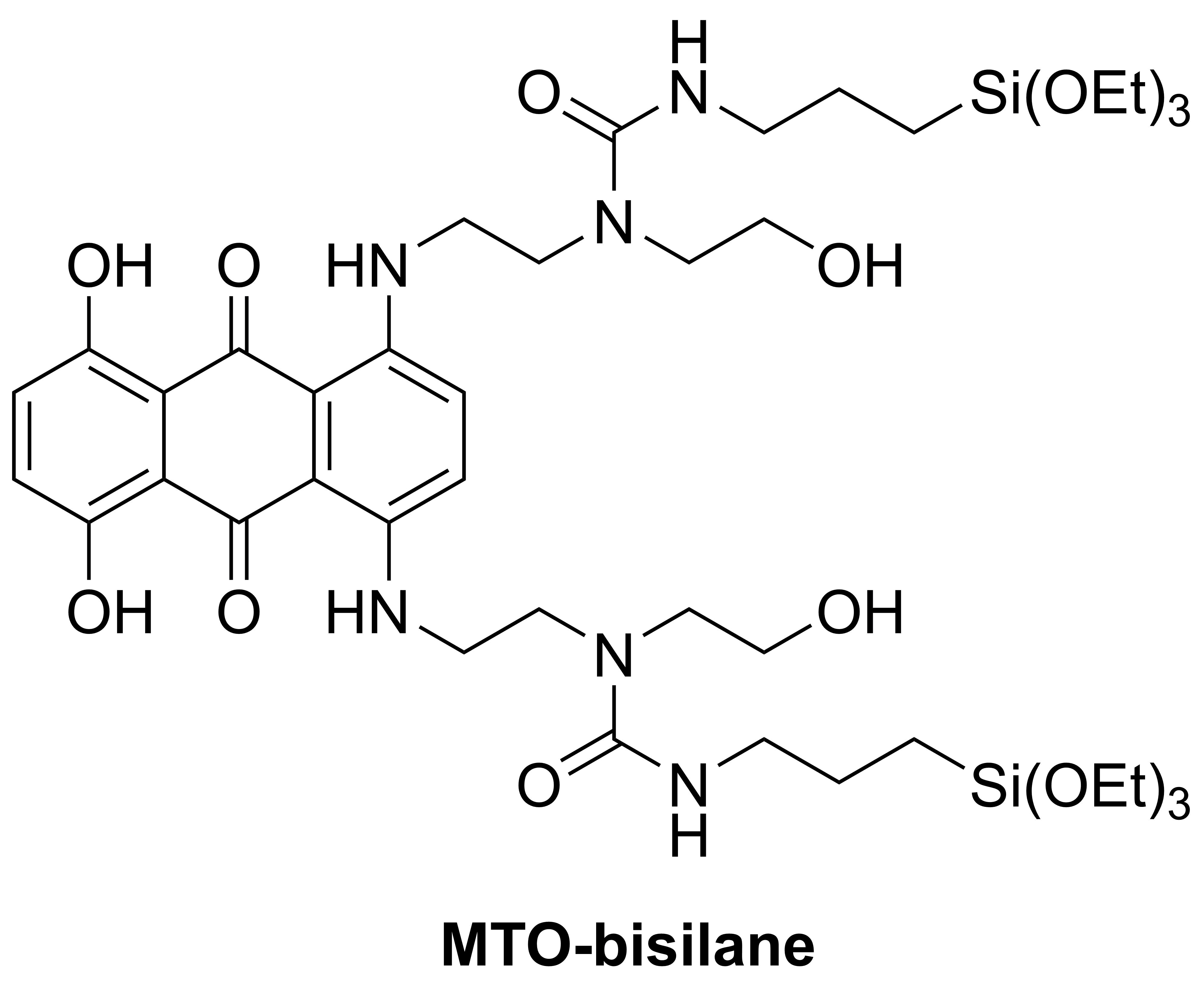


**^1^H NMR (400 Hz, CDCl_3_):** δ 13.20 (s, 2H), 10.04 (t, *J* = 5.0 Hz, 2H), 6.95 (s, 2H), 6.90 (s, 2H), 6.01 (t, *J* = 5.0 Hz, 2H), 4.33 (s, 2H), 3.83−3.77 (m, 16H), 3.53−3.49 (m, 4H), 3.46−3.41 (m, 8H), 3.21−3.16 (m, 4H), 1.66−1.58 (m, 4H), 1.21 (t, *J* = 7 Hz, 18 H), 0.66−0.62 (m, 4H). **^13^C RMN (100 Hz, CDCl_3_):** δ 184.3, 159.9, 155.2, 146.5, 124.4, 123.7, 115.1, 108.4, 77.5, 77.2, 76.8, 62.2, 58.6, 51.7, 48.4, 43.7, 41.3, 23.6, 18.4, 7.9. **ESI MS [M+Na]^+^** = 961.4.


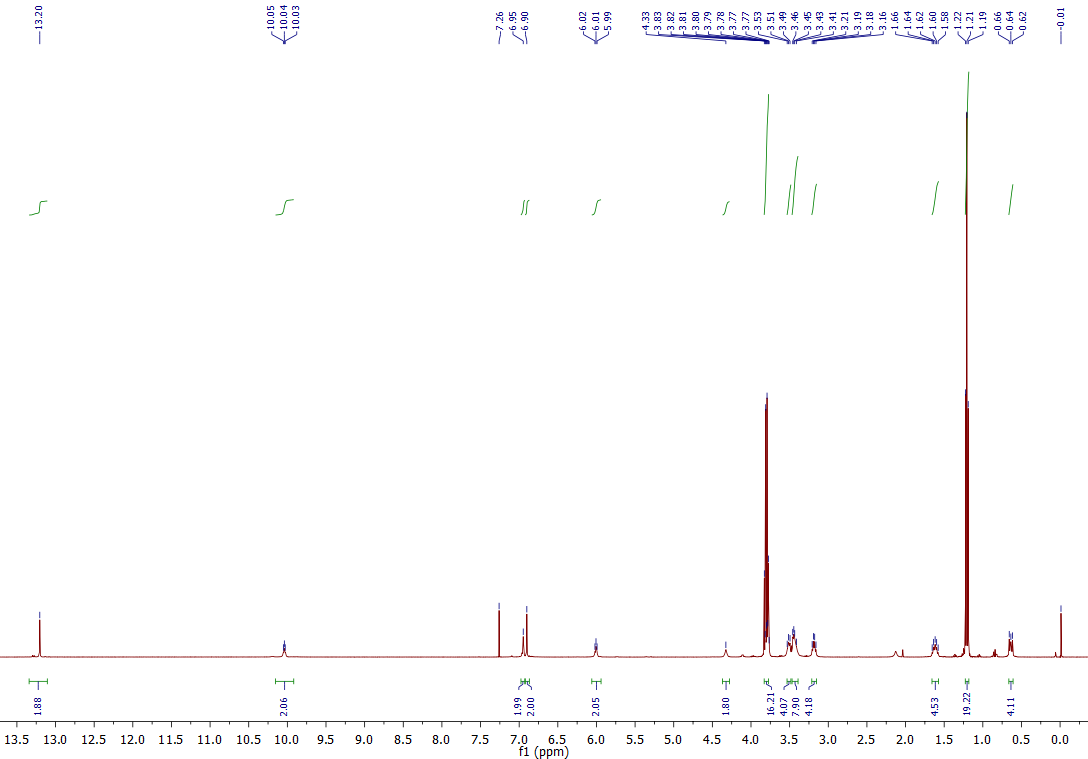


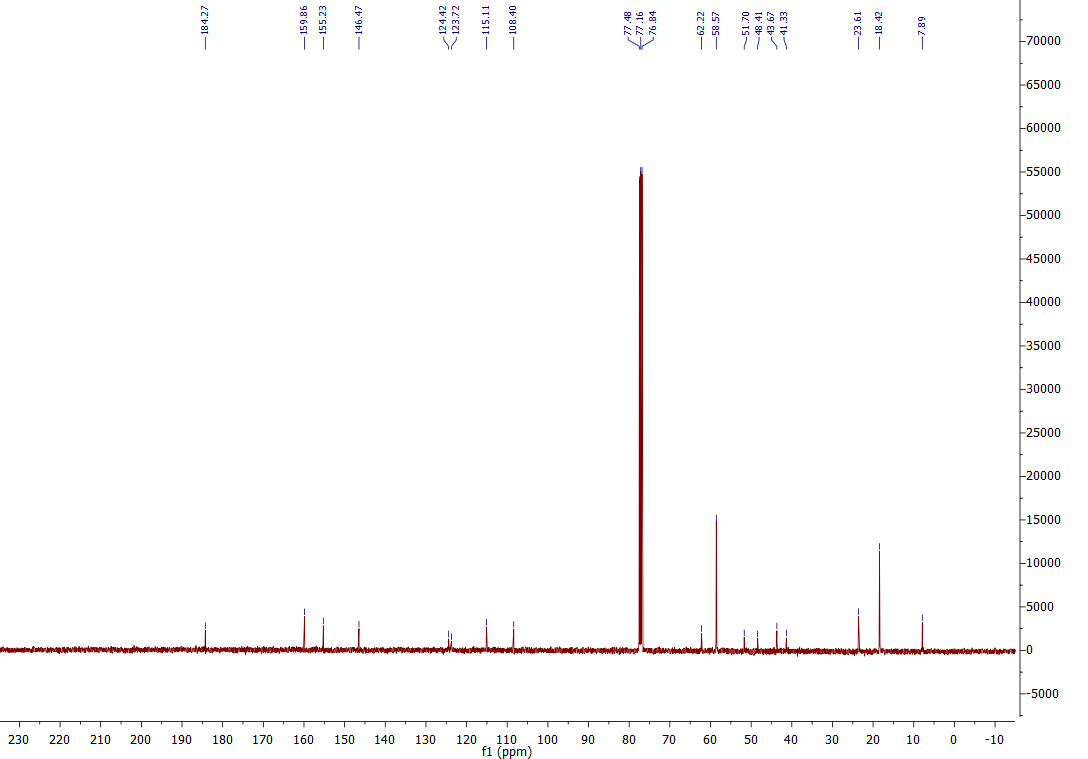


**Figure S1.** ^1^H NMR and ^13^C NMR spectra of MTO-bis-organosilane in CDCl_3_.


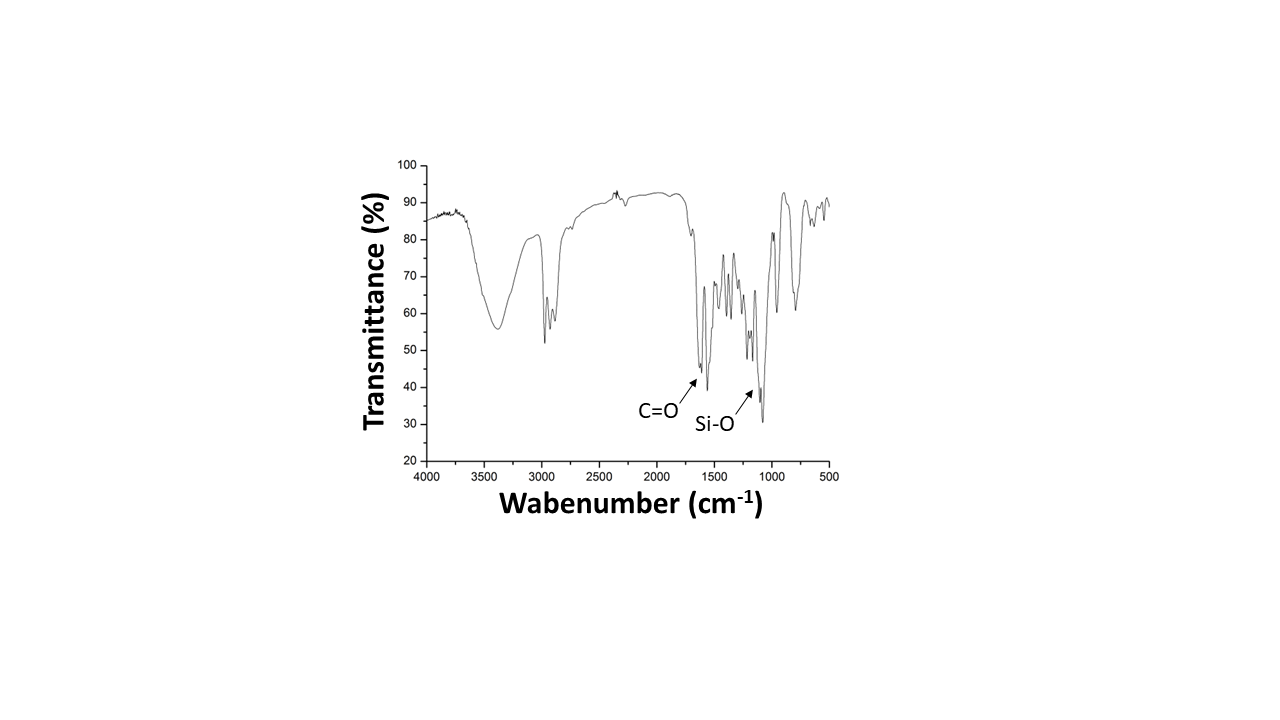


**Figure S2.** FTIR measurements of MTO-bis-organosilane.


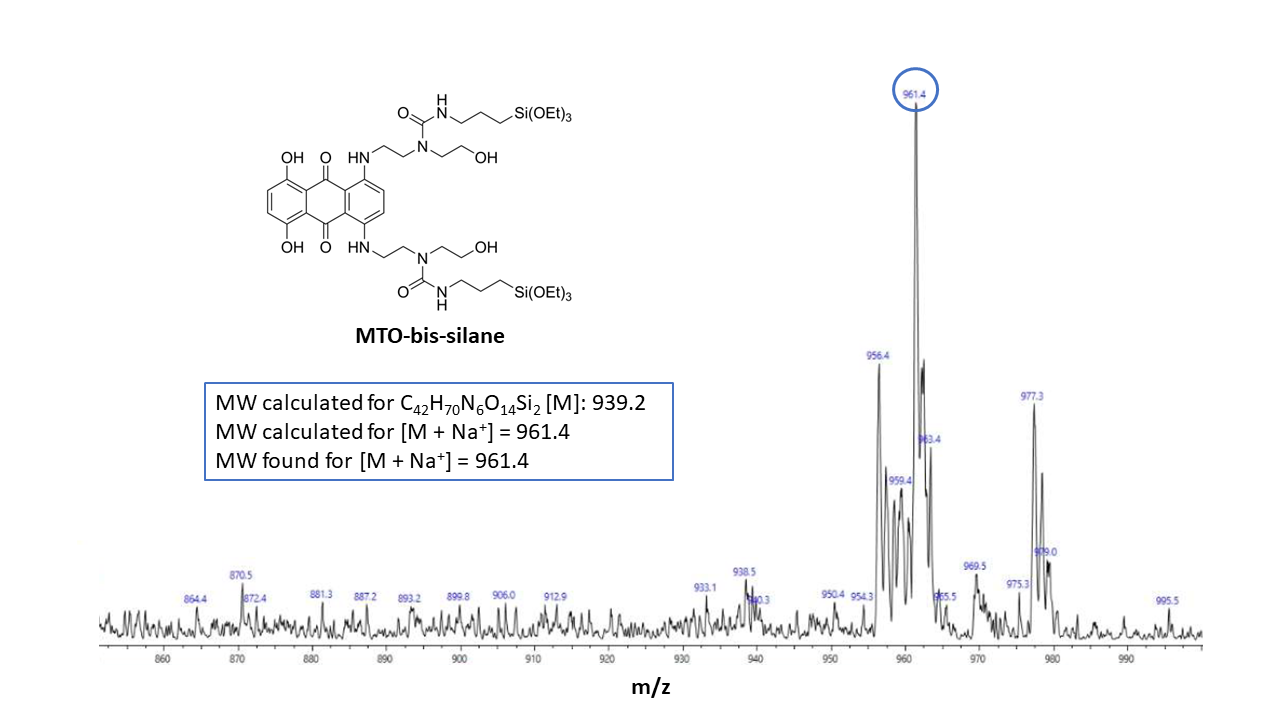


**Figure S3.** ESI-Mass spectrum of MTO-bis-organosilane.

**Characterization of the MSNs (reference material)**

**
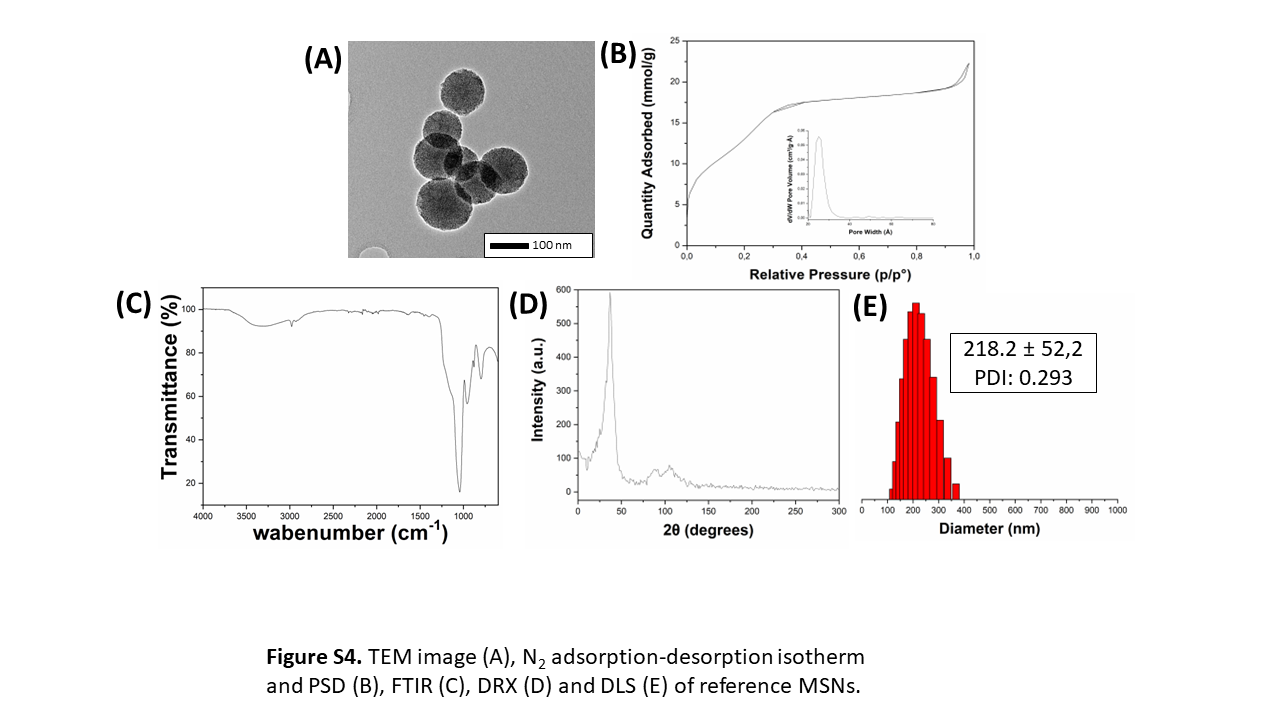
**

**Figure S4.** TEM image (A), N_2_ adsorption-desorption isotherm and PSD inset (B), FTIR (C), DRX (D) and DLS (E) of reference MSNs.

**Optimization procedure of pure MTO NPs (MTO-100@MOD)**

In the first attempt to synthesize a purely organic MOD, we followed the procedure for the reference MSNs using the bis-organosilane derivative of MTO as the only silica source. In order to evaluate the effect of the introduction of MTO-bis-organosilane instead of TEOS, no conditions respecting the protocol for obtaining MSNs were modified, excepting the use of the bis-organosilane instead of TEOS (SI Table S1, Entry 1). For every equivalent of TEOS subtracted, we added 0.5 equivalents of the bis-organosilane derivative of MTO (since the silicon relation between TEOS and MTO-bis-organosilane is 2:1). In this way, we performed the reaction using 1.97 mmol of MTO-bis-organosilane or 3.94 mmol of silicon. Once dissolved in EtOH (9.5 mL), the solution was added dropwise over a mixture of CTAB (0.44 mmol) in MilliQ water at pH = 12 (69 mL of ultrapure water, 820 µL of NaOH 1M). The reaction was stirred for 2 hours at 82 °C and the NPs were filtered off, washed with H_2_O and EtOH and dried under vacuum.

TEM images of the as-made material (MTO-100@MOD-1) showed particles with heterogenous morphologies and sizes (between 50 nm and 3 μm, Figure S5). This may be due to the poor solubility of the MTO-bis-silane in water. The small amount of MTO-bis-silane dissolved in the reaction mixture in the first moment, would create few nucleation points, that would become bigger as more MTO-bis-silane gets dissolved. Since the size of the nanoparticles is critical for their employment as drug delivery systems, we optimized the synthesis by changing several parameters in order to obtain NPs with diameters under 100 nm. The different experiments performed are summarized in table S1.

**Table S1.** Synthesis conditions of NPs using MTO-bis-organosilane as the only source of silica (MTO-100@MODs).

| Entry | Material | Ratio  EtOH : H_2_O | [MTO-bis-organosilane] (mM) | [NaOH] (mM) |
| --- | --- | --- | --- | --- |
| 1 | MTO-100@MOD-1 | 1 : 7 | 2.5 | 10 |
| 2 | MTO-100@MOD-2 | 1 : 4 | 2.5 | 10 |
| 3 | MTO-100@MOD-3 | 1 : 4 | 0.58 | 10 |
| 4 | MTO-100@MOD-4 | 1 : 4 | 0.58 | 6 |
| 5 | MTO-100@MOD-5 | 1 : 2 | 0.58 | 6 |
| 6^a^ | MTO-100@MOD | 1 : 2 | 0.58 | 6 |

^a^ The MTO-bis-silane was carefully purified.

We hypothesized that the addition of more ethanol to the reaction mixture would be beneficial as it would ensure a better solubility of the MTO-bis-silane. For this reason, we tested the reaction increasing the ratio of EtOH : H_2_O from 1 : 7 to 1 : 4 (Table S1, entry 2). The TEM micrographs of these materials (MTO-100@MOD-2) showed spherical NPs with different sizes (Figure S5). Additionally, the images showed agglomeration of the particles with neck growth between them, which could explain the particle diameter measured by DLS (1228 ± 442 nm).

The large dimensions of the particles could be due to the bigger size of MTO-bis-organosilane compared to TEOS. For this reason, the next change that we performed in the reaction conditions was to reduce the concentration of the MTO-bis-organosilane from 2.5 mM to 0.58 mM (MTO-100@MOD-3) (Table S1, entry 3). The dilution of the reaction could ensure a slower polymerization, yielding more homogeneous NPs. The TEM micrographs showed spherical and nanometre-sized NPs (Figure S5), as DLS measures confirmed (741 ± 67 nm). However, the yield of the reaction decreased. When the rate of hydrolysis is too fast, too many nucleation points are created, producing a colloid with small soluble chains of the precursor and NPs that results in a decreased yield.^[1,2]^ The presence of small unreacted alkoxysilanes may contribute to neck formation.

So, with the aim of reducing the rate of hydrolysis of MTO-bis-organosilane and TEOS to increase the yield of the reaction, further parameters of the reaction were changed. First, the concentration of the base in the reaction mixture was decreased from 10 to 6 mM (Table S1, entry 4). The TEM micrographs showed NPs with a more defined morphology (MTO-100@MOD-4, Figure S5). However, the neck growth and yield were still an issue, as shown by the DLS measurement of the hydrodynamic diameter of the material (552 ± 159 nm), so we continued with the optimization of the reaction conditions.

Next, the ratio of EtOH : H_2_O was reduced to 1 : 2. The increase in EtOH content is related to a slowdown of the hydrolysis rate.^[3,4]^ In truth, the yield of the reaction was increased, but the problem of the neck formation between the NPs persisted (MTO-100@MOD-5), as can be seen in the TEM micrographs (Figure S5).

In the MTO-bis-organosilane synthesis, an excess of 3-(triethoxysilyl)propylisocyanate is used. The presence of impurities could be contributing to the formation of necks between the nanoparticles if the bis-organosilane is not carefully purified. For this reason, we purified the MTO-bis-organosilane meticulously by performing several washings of the solid with petroleum ether. Finally, we were able to obtain dispersed and spherical NPs with homogeneous size (MTO-100@MOD), as it can be observed in the TEM images (Figure S5).


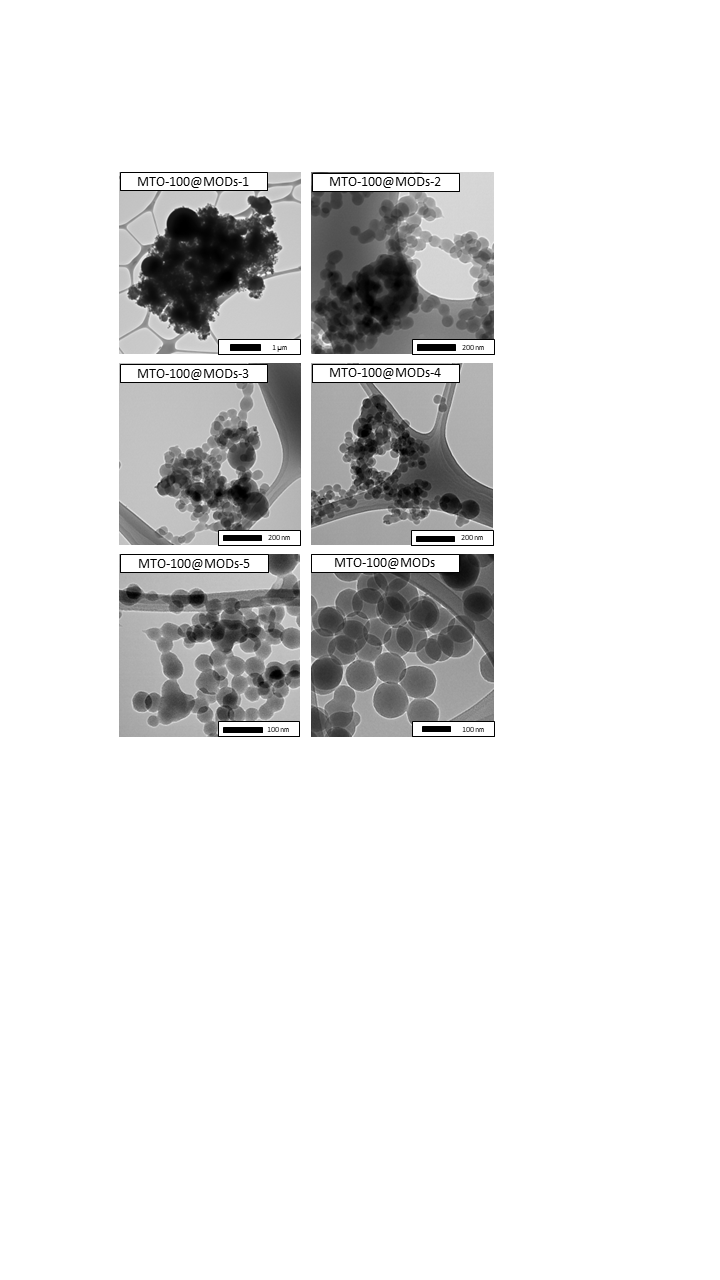


**Figure S5.** TEM micrographs of the NPs using 100% of MTO-bis-organosilane (MTO-100@NPs) synthesized according to the reaction conditions in Table S1.

**Characterization of MTO-100@MOD, MTO-50@MOD, MTO-20@MOD and MTO-10@MOD**

**

**

**Figure S6.** Characterization of MTO@MODs by thermogravimetric analysis.

**Table S2**. Expected values for the organic content in the different MODs and the obtained value using TGA in brackets.

| MOD material | % Organic content |
| --- | --- |
| 100-MTO@MOD | 79 (79) |
| 50-MTO@MOD | 57 (56) |
| 20-MTO@MOD | 31 (31) |
| 10-MTO@MOD | 18 (18) |

**Table S3**. Expected values for the % of C and N in the different MODs and the obtained value using EA in brackets.

| MOD material | % of C | % of N |
| --- | --- | --- |
| 100-MTO@MOD | 50.3 (46.6) | 11.7 (10.9) |
| 50-MTO@MOD | 36.5 (32.7) | 8.5 (7.6) |
| 20-MTO@MOD | 20.0 (19.6) | 4.7 (3.9) |
| 10-MTO@MOD | 11.4 (11.5) | 2.7 (2.0) |


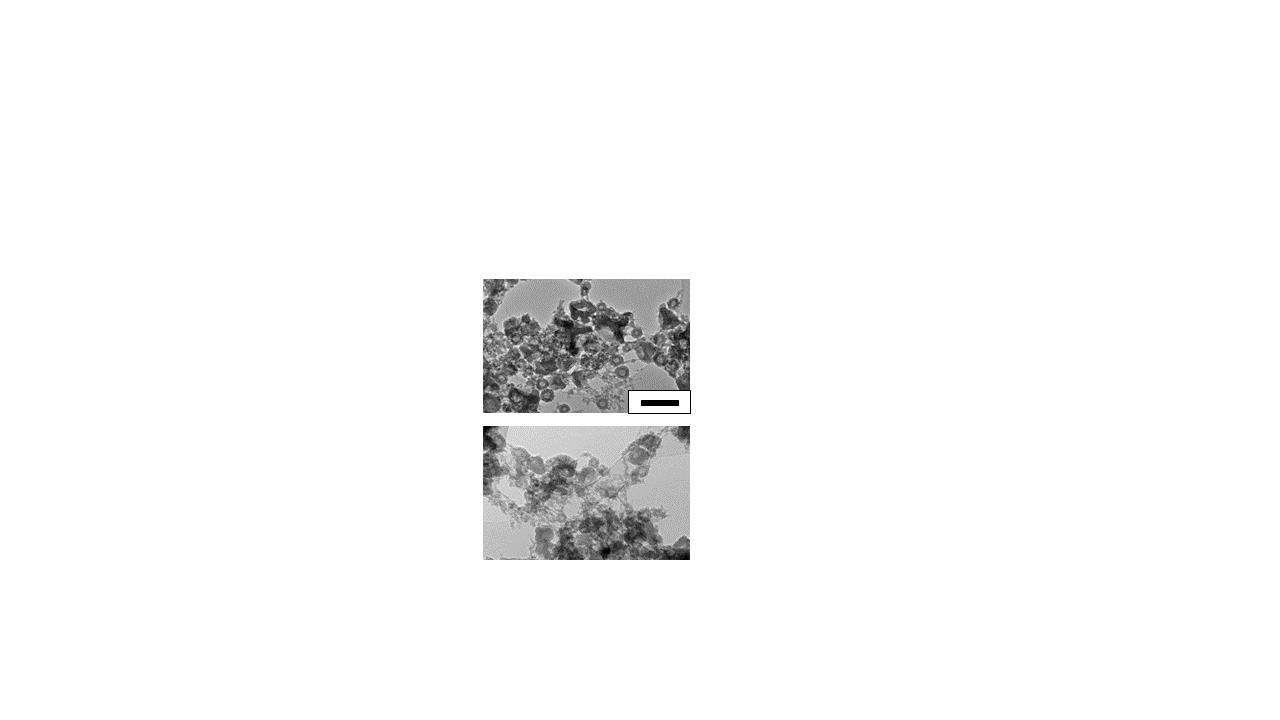


**Figure S7.** TEM micrographs of a material synthesized following MTO-100@MOD procedure but without the addition of surfactant. Scale bar at 200 nm.


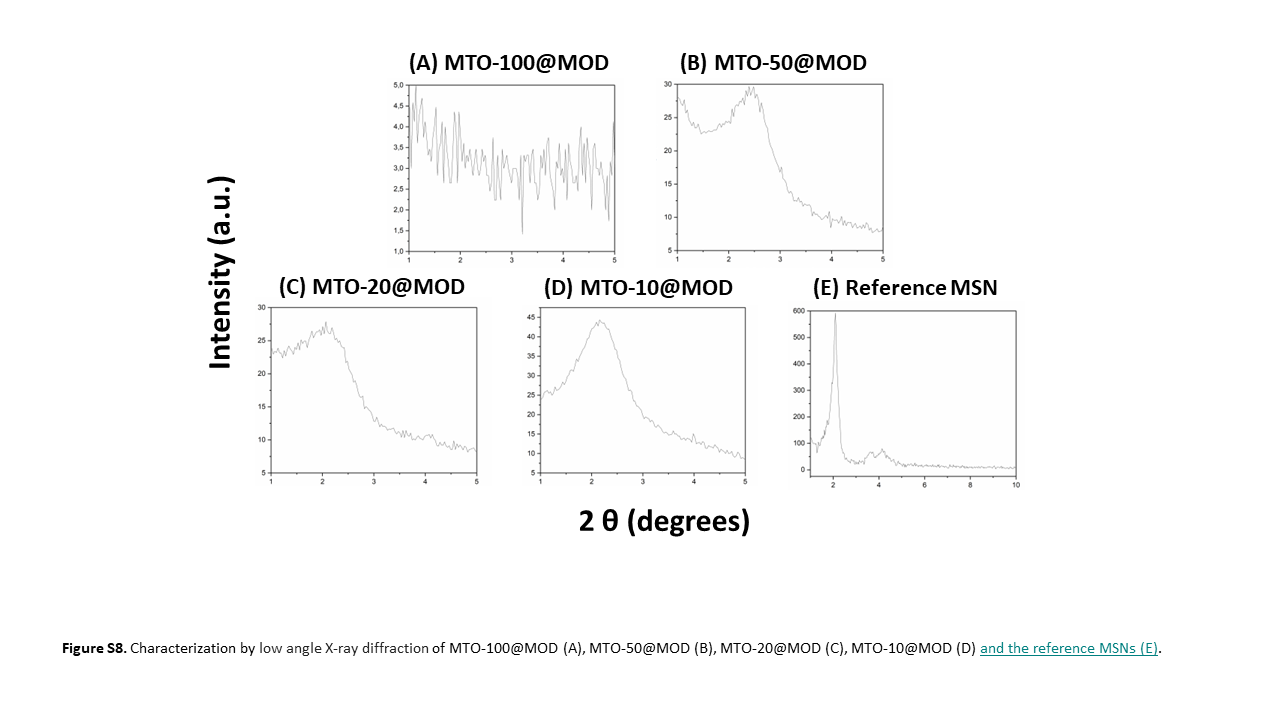


**Figure S8.** Characterization by low angle X-ray diffraction of MTO-100@MOD (A), MTO-50@MOD (B), MTO-20@MOD (C), MTO-10@MOD (D) and the reference MSNs (E).

**Table S4**. Percentage of Q and T sites from CP/MAS ^29^Si MAS NMR spectra of MTO-100@MOD, MTO-50@MOD, MTO-20@MOD and MTO-10@MOD.

|  | % Q sites | % T sites |
| --- | --- | --- |
| MTO-100@MOD | 0 | 100 |
| [MTO-50@MOD](mailto:MTO-50@MODs) | 38 | 62 |
| [MTO-20@MOD](mailto:MTO-20@MODs) | 74 | 26 |
| [MTO-10@MOD](mailto:MTO-10@MODs) | 84 | 16 |


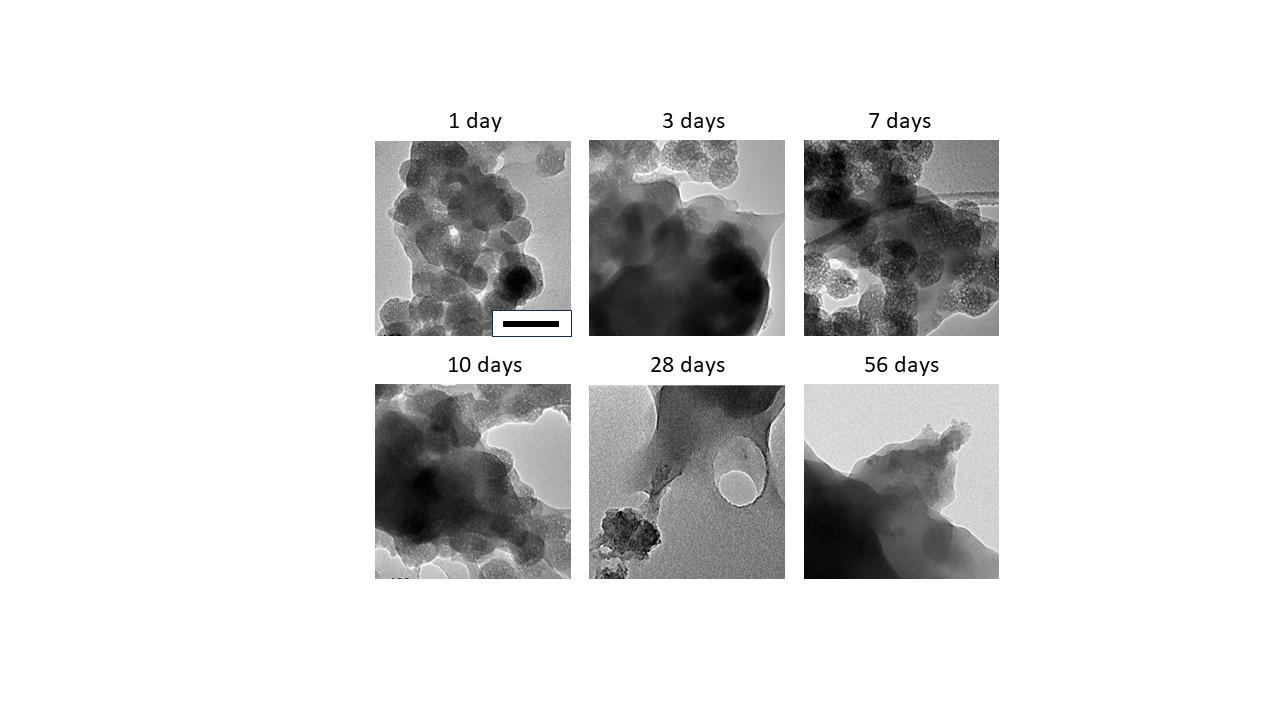


**Figure S9.** TEM images of MTO-20@MOD exposed to human serum through the time, at 37 °C and 1400 rpm. Scale bar at 100 nm.


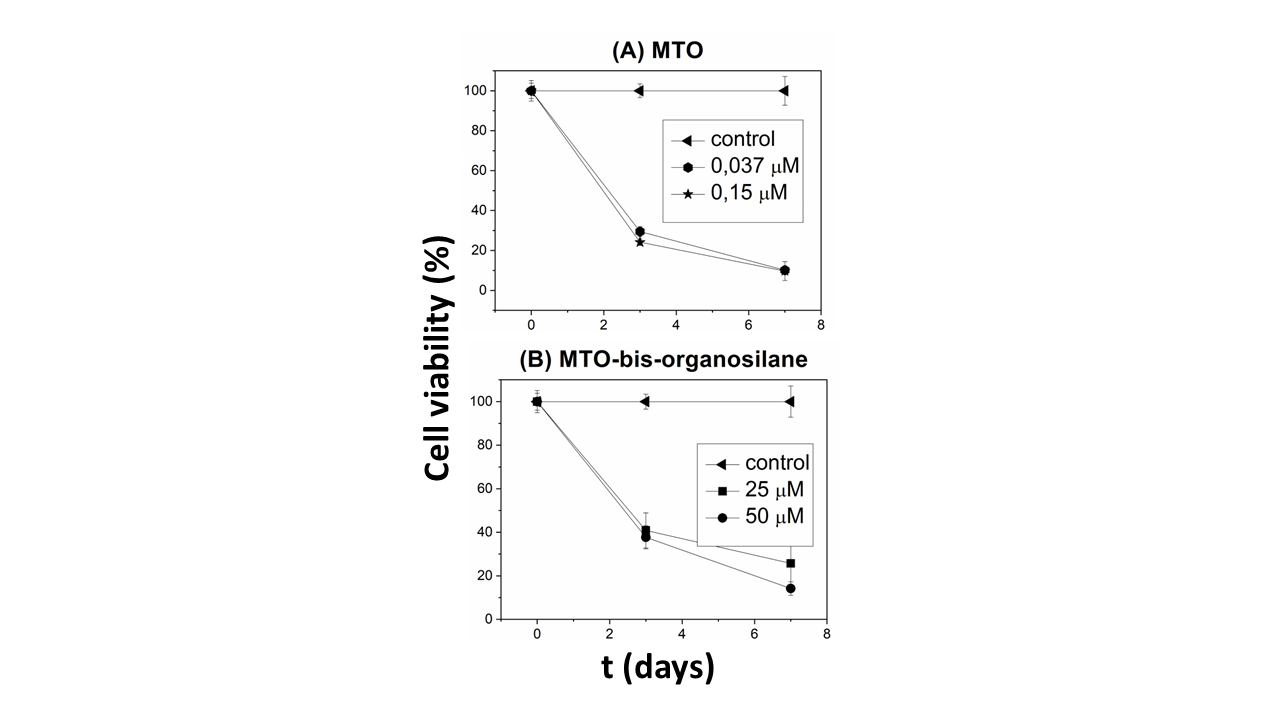


**Figure S10**. Cell viability loss of MCF-7 cells through MTT assay at different concentrations of MTO (A) and MTO-bis-organosilane (B).





**Figure S11**. Cell viability of MCF-7 through MTT assay treated with the reference material compared to the negative control.


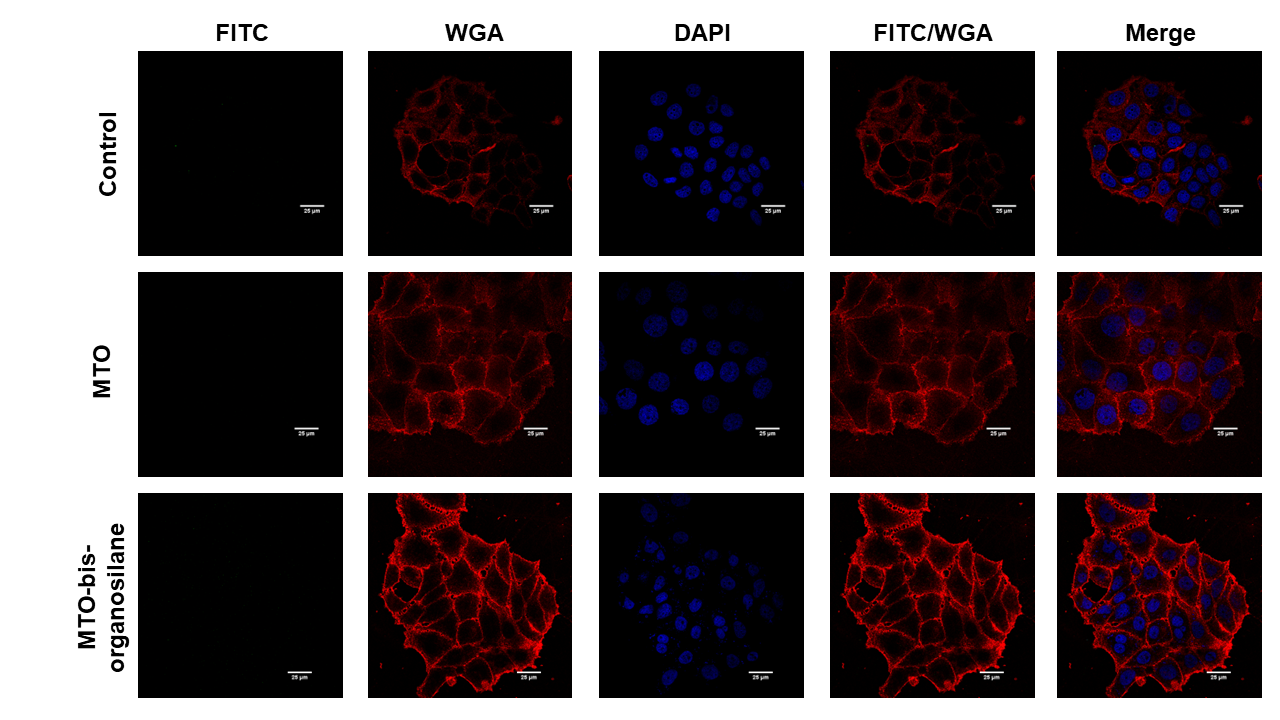


**Figure S12.** Confocal imaging (single plane) of MCF-7 cells incubated with MTO (0.15 μM) and MTO-bis-organosilane (50 µM). Control cells were cultured without NPs nor drug or prodrug. Cell membranes were stained with WGA (red) and nuclei with DAPI (blue).


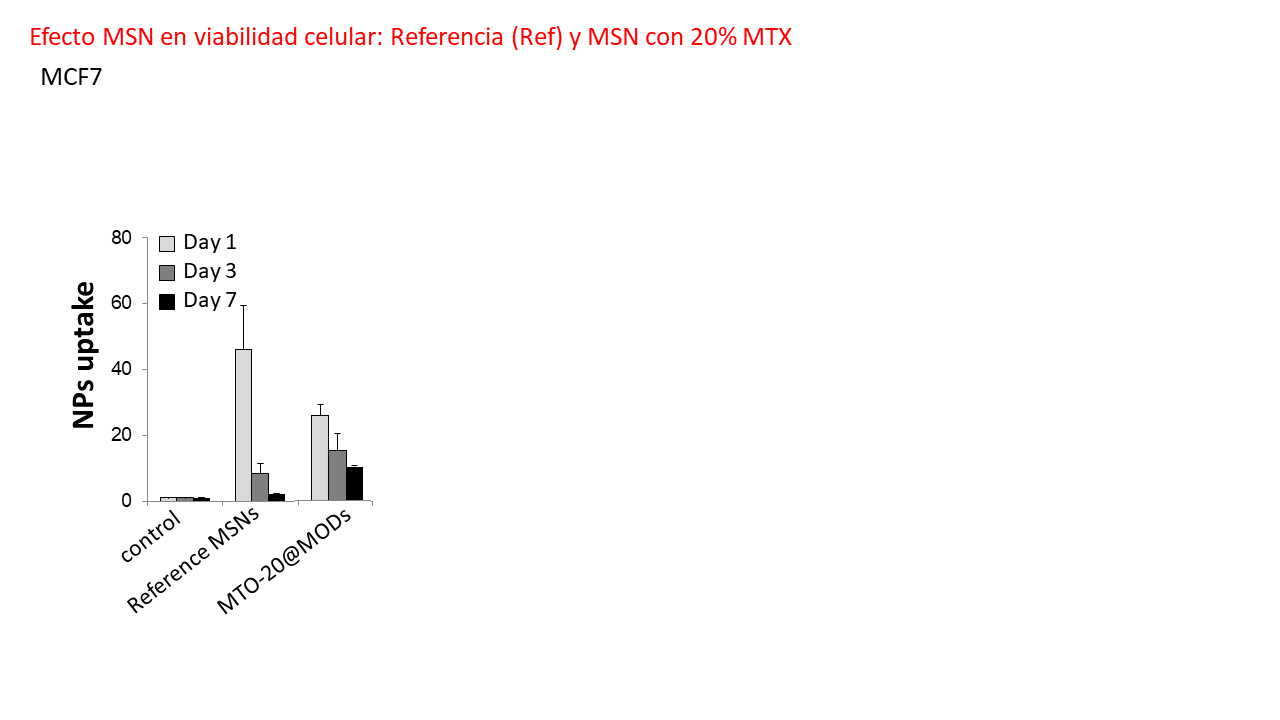


**Figure S13.** Cellular uptake of MCF-7 cells of the reference MSNs and MTO-20@MODs (at 10 μg mL^-1^).


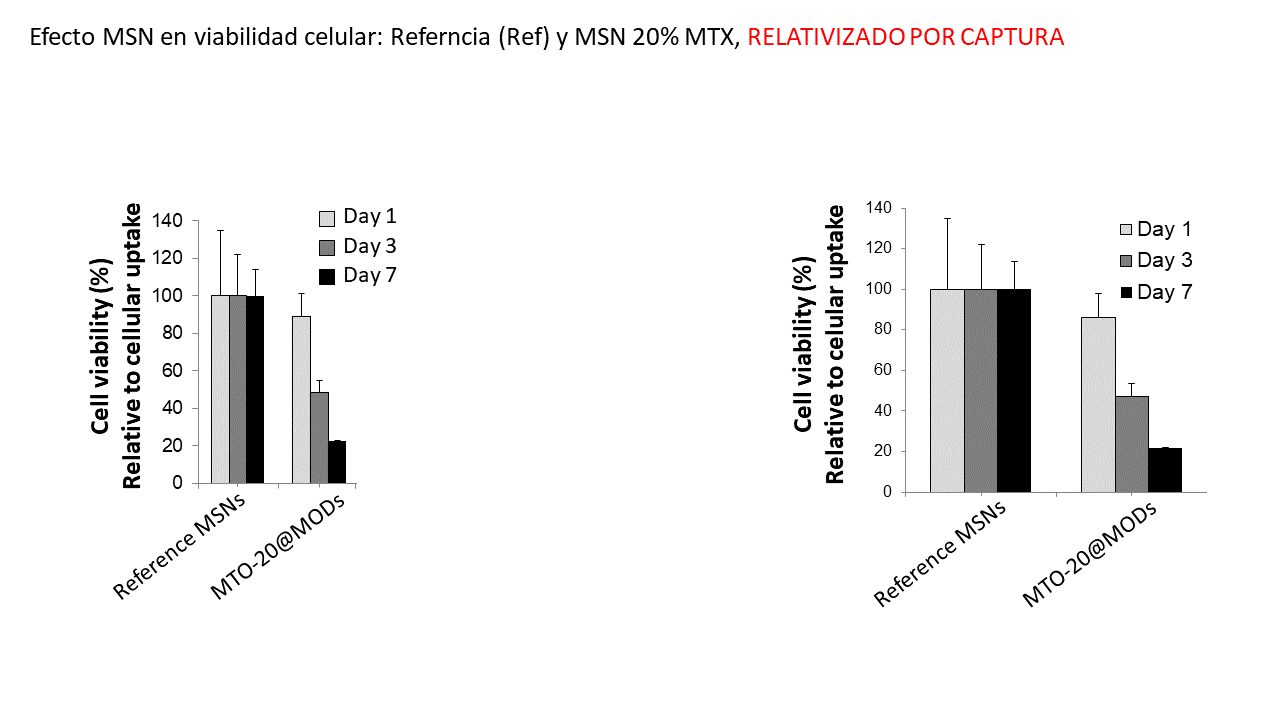


**Figure S14.** Cell viability relative to cellular uptake of MCF-7 cells treated with the reference MSNs and MTO-20@MODs (at 10 μg mL^-1^) analysed by flow cytometry.


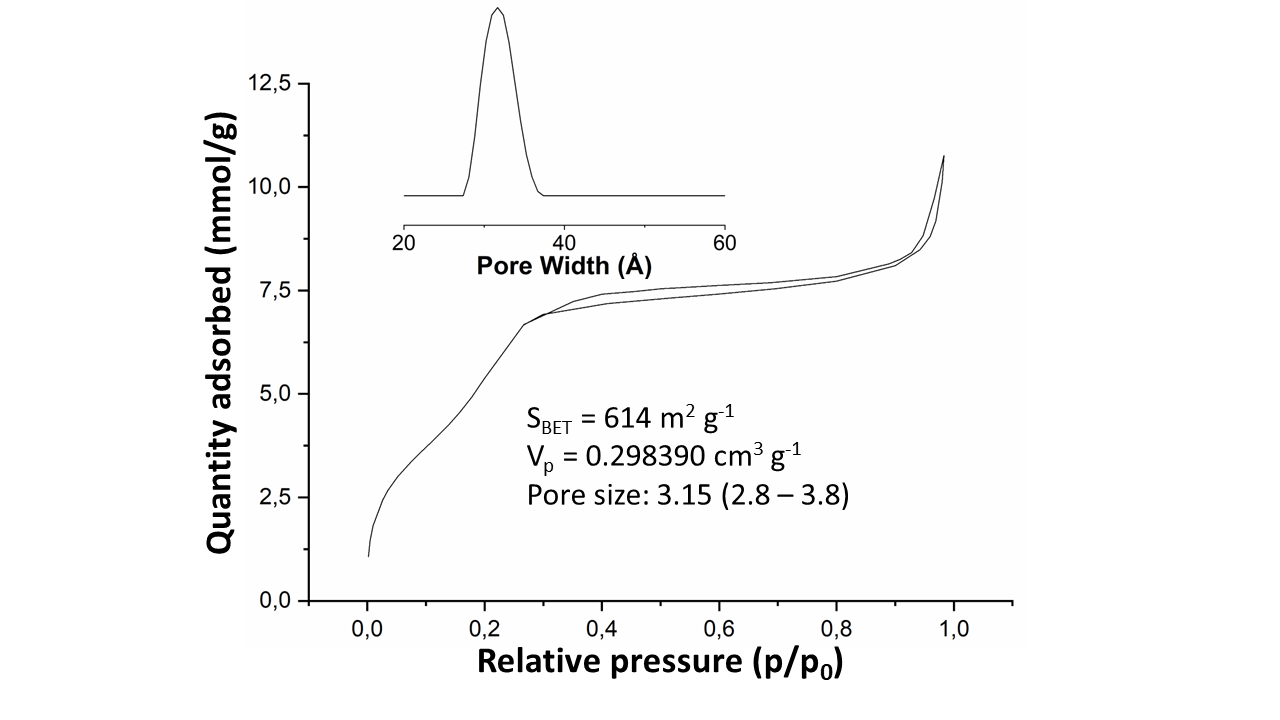


**Figure S15.** N_2_ adsorption-desorption isotherms of magnetic MTO-20@MOD, PSD in inset.


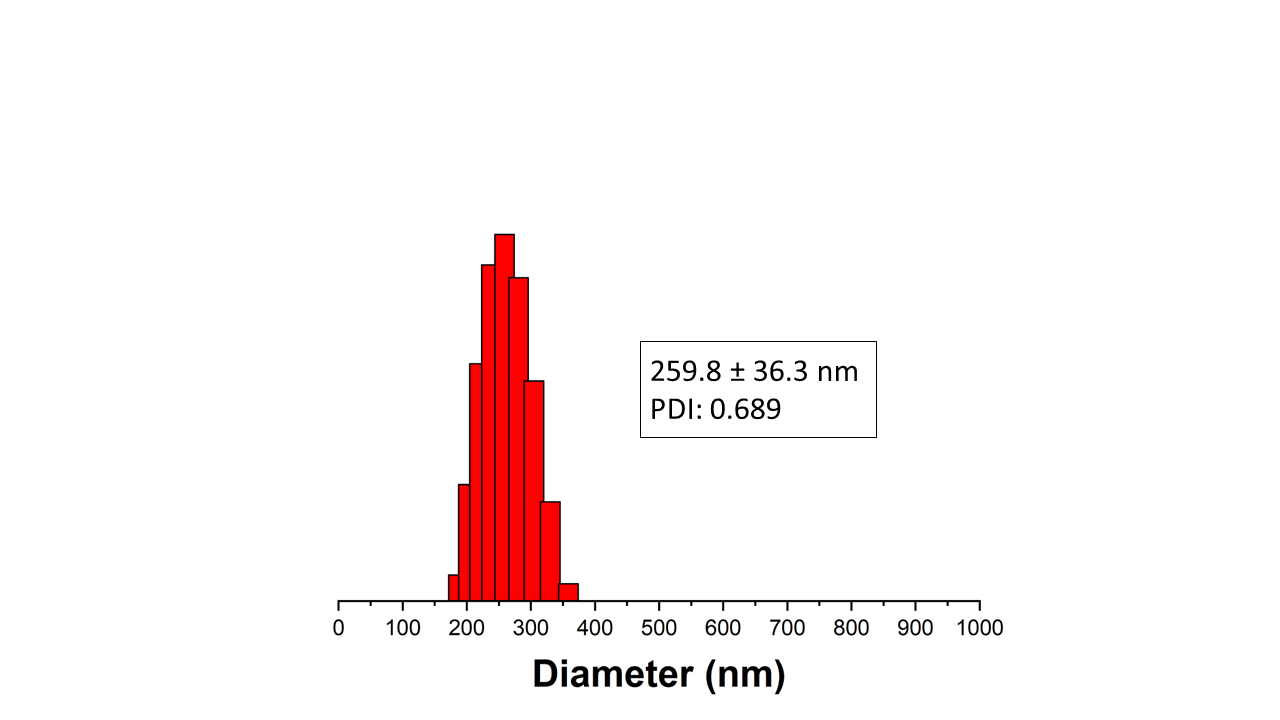


**Figure S16.** DLS measurement of magnetic MTO-20@MOD.





**Figure S17.** Effect of magnetic MTO-20@MOD in H_2_O_2_ concentration over time.


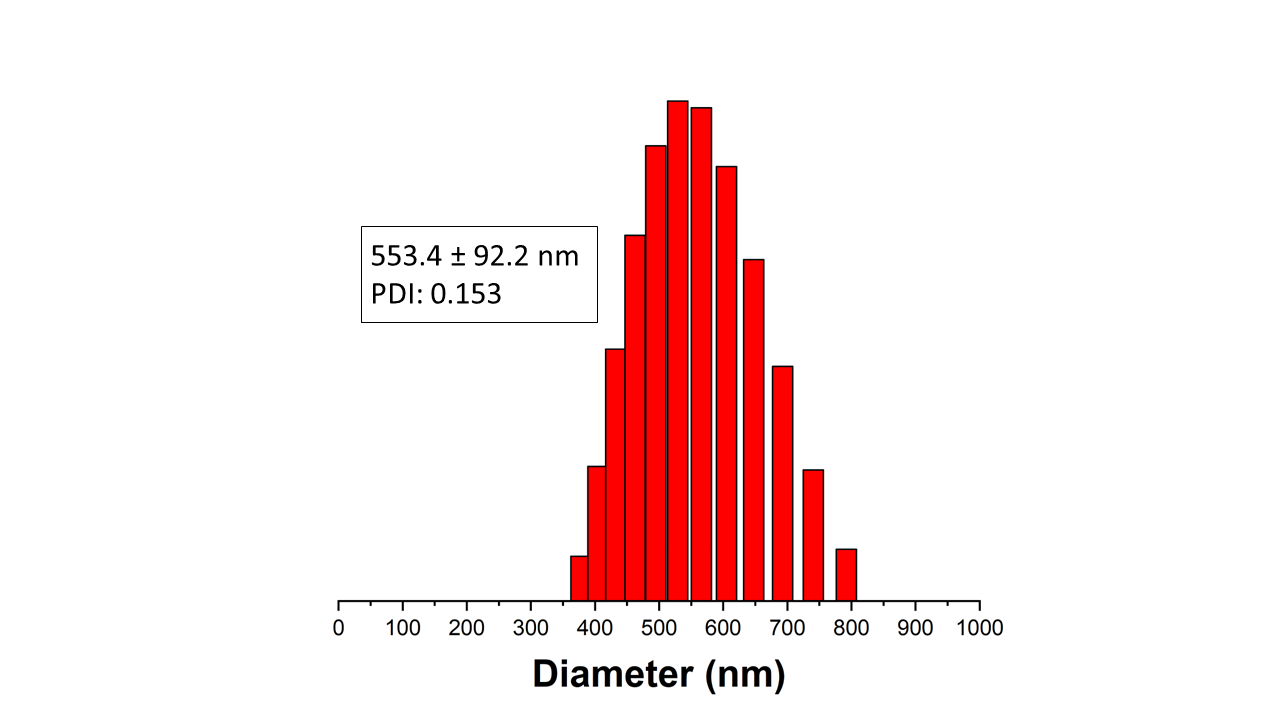


**Figure S18.** DLS measurement of magnetic MTO-100@MOD.

**Bibliography**

[1] C. Vidaurre-Agut, E. Rivero-Buceta, E. Romaní-Cubells, A. M. Clemments, C. D. Vera-Donoso, C. C. Landry, P. Botella, *ACS Omega* **2019**, *4*, 8852.

[2] T. L. Riss, R. A. Moravec, A. L. Niles, H. A. Benink, T. J. Worzella, L. Minor, 25.

[3] Y. Yang, S. Bernardi, H. Song, J. Zhang, M. Yu, J. C. Reid, E. Strounina, D. J. Searles, C. Yu, *Chem. Mater.* **2016**, *28*, 704.

[4] H. Yamada, C. Urata, S. Higashitamori, Y. Aoyama, Y. Yamauchi, K. Kuroda, *ACS Appl. Mater. Interfaces* **2014**, *6*, 3491.
